# Supplementary material for: Antimicrobial Chlorinated 3-Phenylpropanoic Acid Derivatives from the Red Sea Marine Actinomycete Streptomyces coelicolor LY001
Source: Mar Drugs. 2020 Aug 27;18(9):450. doi: 10.3390/md18090450 (PMC7551466; doi:10.3390/md18090450)
Supplement: Supplementary file 1 [file marinedrugs-18-00450-s001.pdf]

Supplementary Information

Figure S1. ^{1}H NMR spectrum of 3-(3,5-dichloro-4-hydroxyphenyl)propanoic acid (**1**) ($CDCl_3$) S1

Figure S2. ^{13}C NMR spectrum of 3-(3,5-dichloro-4-hydroxyphenyl)propanoic acid (**1**) ($CDCl_3$) S1

Figure S3. ^{1}H-^{1}H COSY NMR spectrum of 3-(3,5-dichloro-4-hydroxyphenyl)propanoic acid (**1**) ($CDCl_3$) S2

Figure S4. HSQC spectrum of 3-(3,5-dichloro-4-hydroxyphenyl)propanoic acid (**1**) ($CDCl_3$) S2

Figure S5. HMBC spectrum of 3-(3,5-dichloro-4-hydroxyphenyl)propanoic acid (**1**) ($CDCl_3$) S3

Figure S6. HRESIMS spectrum of 3-(3,5-dichloro-4-hydroxyphenyl)propanoic acid (**1**) S3

Figure S7. ^{1}H NMR spectrum of 3-(3,5-dichloro-4-hydroxyphenyl)propanoic acid methyl ester (**2**) ($CDCl_3$). S4

Figure S8. ^{13}C NMR spectrum of 3-(3,5-dichloro-4-hydroxyphenyl)propanoic acid methyl ester (**2**) ($CDCl_3$) S4

Figure S9. ^{1}H-^{1}H COSY 3-(3,5-dichloro-4-hydroxyphenyl)propanoic acid methyl ester (**2**) ($CDCl_3$) S5

Figure S10. HSQC spectrum 3-(3,5-dichloro-4-hydroxyphenyl)propanoic acid methyl ester (**2**) ($CDCl_3$) S5

Figure S11. HMBC spectrum 3-(3,5-dichloro-4-hydroxyphenyl)propanoic acid methyl ester (**2**) ($CDCl_3$) S6

Figure S12. HRESIMS spectrum of 3-(3,5-dichloro-4-hydroxyphenyl)propanoic acid methyl ester (**2**) S6

Figure S13. ^{1}H NMR spectrum of 3-(3-chloro-4-hydroxyphenyl)propanoic acid (**3**) ($CDCl_3$) S7

Figure S14. ^{13}C NMR spectrum of 3-(3-chloro-4-hydroxyphenyl)propanoic acid (**3**) ($CDCl_3$) S7

Figure S15. ^{1}H-^{1}H COSY NMR of 3-(3-chloro-4-hydroxyphenyl)propanoic acid (**3**) ($CDCl_3$) S8

Figure S16. HSQC spectrum of of 3-(3-chloro-4-hydroxyphenyl)propanoic acid (**3**) ($CDCl_3$) S8

Figure S17. HMBC spectrum of of 3-(3-chloro-4-hydroxyphenyl)propanoic acid (**3**) ($CDCl_3$) S9

Figure S18. HRESIMS spectrum of 3-(3-chloro-4-hydroxyphenyl)propanoic acid (**3**) S9

Figure S19. ^{1}H NMR spectrum of 3-phenylpropanoic acid (**4**) ($CDCl_3$) S10

Figure S20. ^{13}C NMR spectrum of 3-phenylpropanoic acid (**4**) ($CDCl_3$) S10

Figure S21. ^{1}H-^{1}H COSY NMR spectrum of 3-phenylpropanoic acid (**4**) ($CDCl_3$) S11

Figure S22. HSQC spectrum of 3-phenylpropanoic acid (**4**) ($CDCl_3$) S11

Figure S23. HMBC spectrum of 3-phenylpropanoic acid (**4**) ($CDCl_3$) S12

Figure S24.^{1}H NMR spectrum of *E*-cinnamic acid (**5**) ($CDCl_3$) S12

Figure S25.^{13}C NMR spectrum of *E*-cinnamic acid (**5**) ($CDCl_3$) S13

Figure S26. ^{1}H NMR spectrum of cyclo(L-Phe-*trans*-4-OH-L-Pro) (**6**) ($CDCl_3$) S13

Figure S27. ^{13}C NMR spectrum of cyclo(L-Phe-*trans*-4-OH-L-Pro) (**6**) ($CDCl_3$) S14

Figure S28.^{1}H-^{1}H COSY NMR spectrum of cyclo(L-Phe-*trans*-4-OH-L-Pro) (**6**) ($CDCl_3$) S14

Figure S29. HSQC spectrum of cyclo(L-Phe-*trans*-4-OH-L-Pro) (**6**) ($CDCl_3$) S15

Figure S30. HMBC spectrum of cyclo(L-Phe-*trans*-4-OH-L-Pro) (**6**) ($CDCl_3$) S15

Figure S31. ^{1}H NMR spectrum of cyclo(L-Phe-*cis*-4-OH-D-Pro) (**7**) ($CDCl_3$) S16

Figure S32. ^{13}C NMR spectrum of cyclo(L-Phe-*cis*-4-OH-D-Pro) (**7**) ($CDCl_3$) S16

Figure S33. ^{1}H-^{1}H COSY NMR spectrum of cyclo(L-Phe-*cis*-4-OH-D-Pro) (**7**) ($CDCl_3$) S17

Figure S34. HSQC spectrum of cyclo(L-Phe-*cis*-4-OH-D-Pro) (**7**) ($CDCl_3$) S17

Figure S35. HMBC spectrum of cyclo(L-Phe-*cis*-4-OH-D-Pro) (**7**) ($CDCl_3$) S18

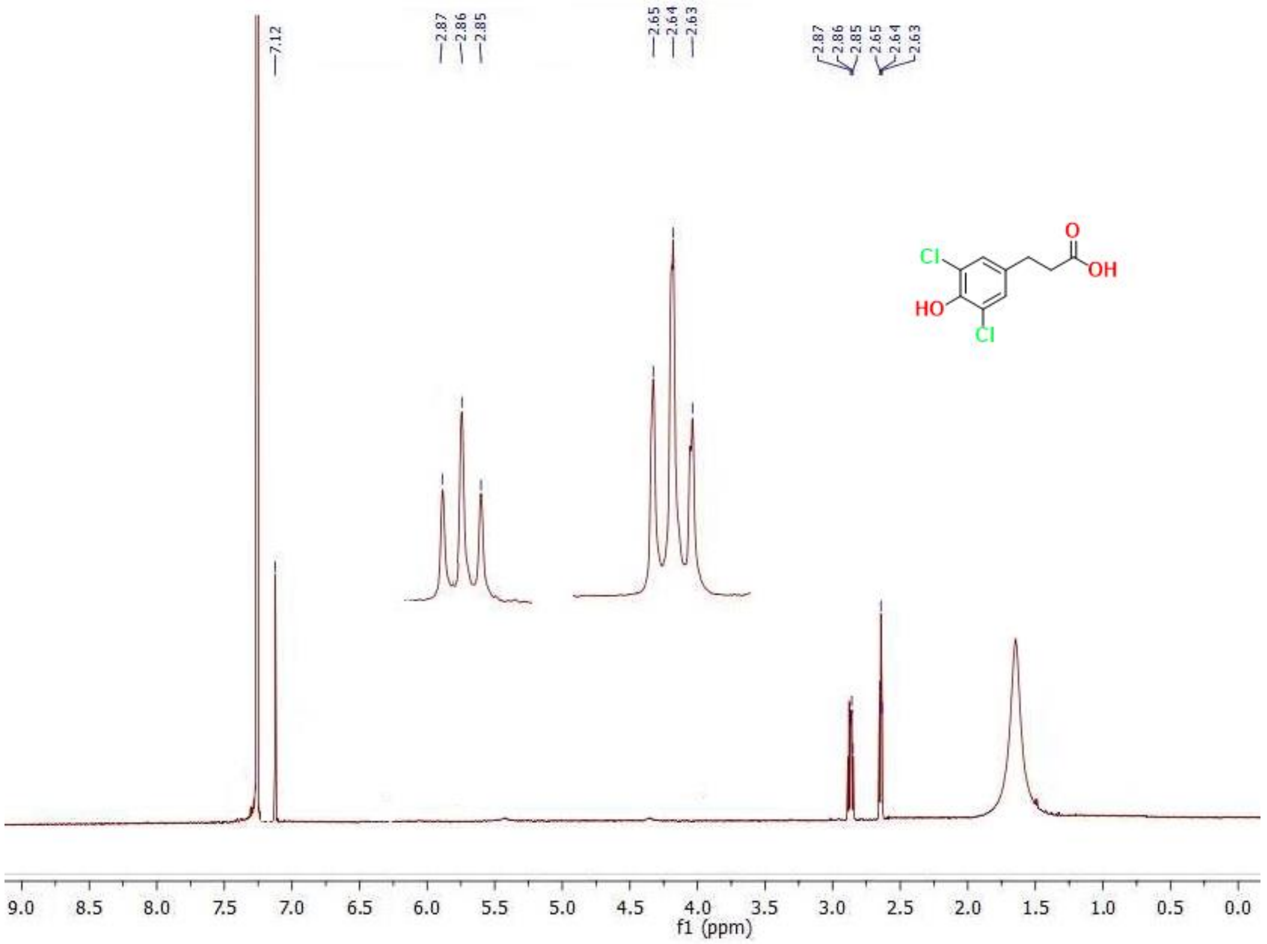

Figure S1. ^{1}H NMR spectrum of 3-(3,5-dichloro-4-hydroxyphenyl)propanoic acid (**1**) ($CDCl_3$)

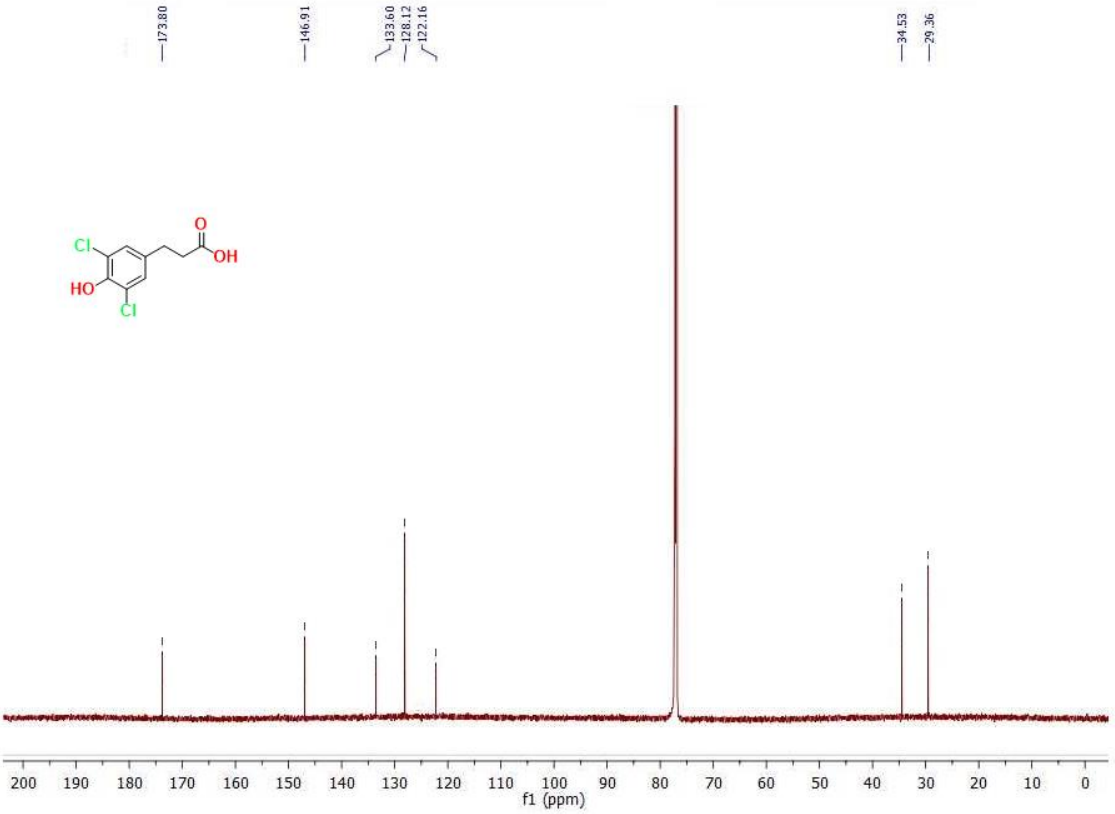

Figure S2. ^{13}C NMR spectrum of 3-(3,5-dichloro-4-hydroxyphenyl)propanoic acid (**1**) ($CDCl_3$).

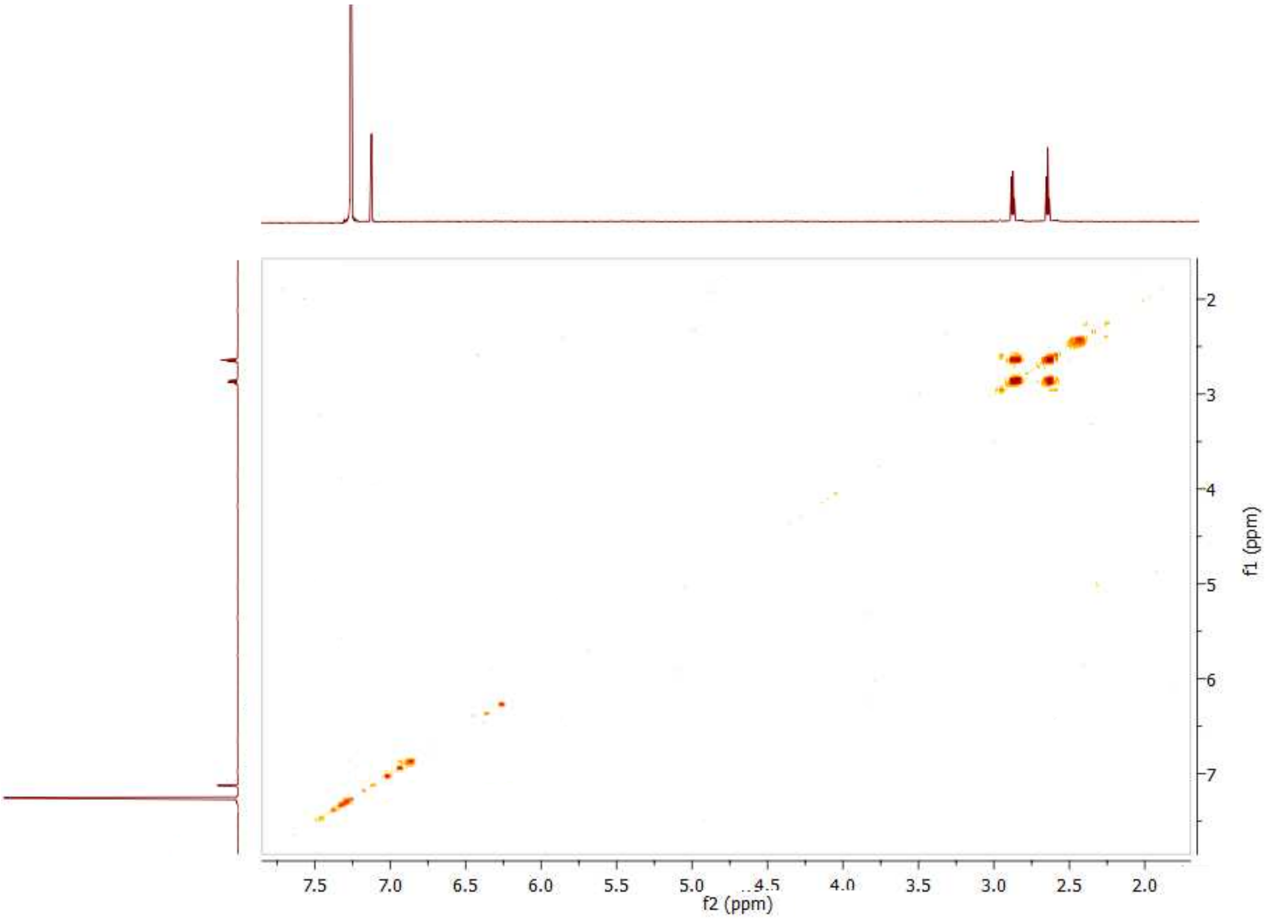

Figure S3. ^{1}H-^{1}H COSY spectrum of 3-(3,5-dichloro-4-hydroxyphenyl)propanoic acid (**1**) ($CDCl_3$).

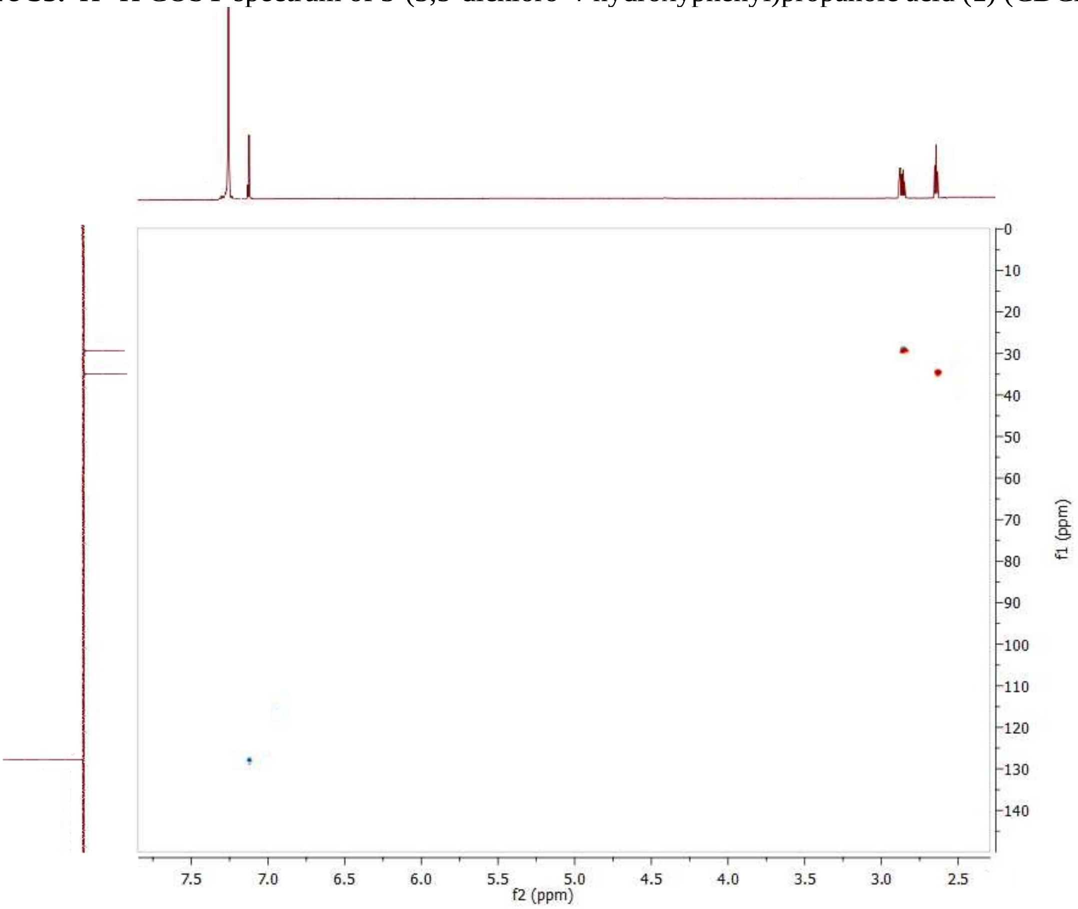

Figure S4. HSQC spectrum of 3-(3,5-dichloro-4-hydroxyphenyl)propanoic acid (**1**) ($CDCl_3$).

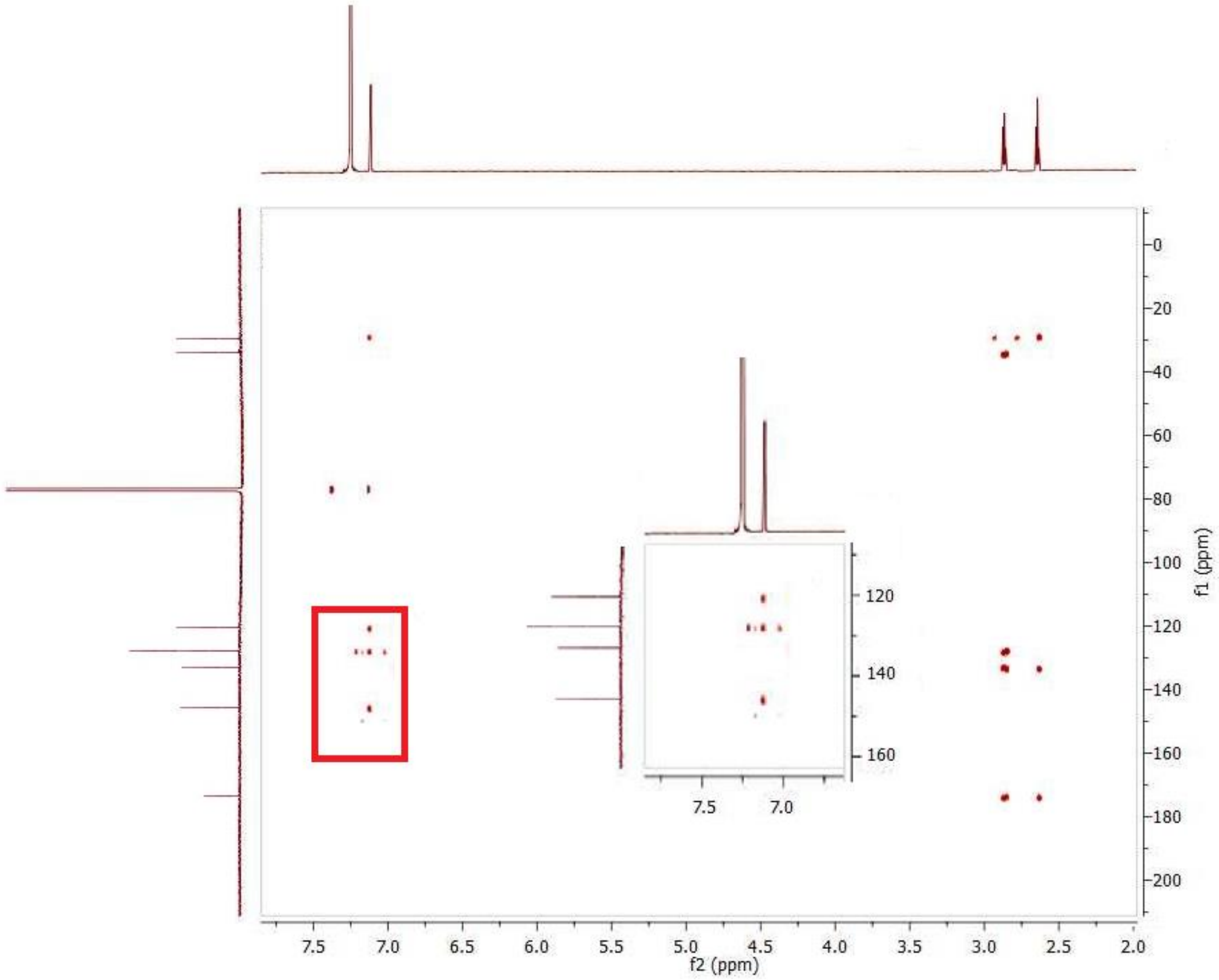

Figure S5. HMBC spectrum of 3-(3,5-dichloro-4-hydroxyphenyl)propanoic acid (**1**) ($CDCl_3$).

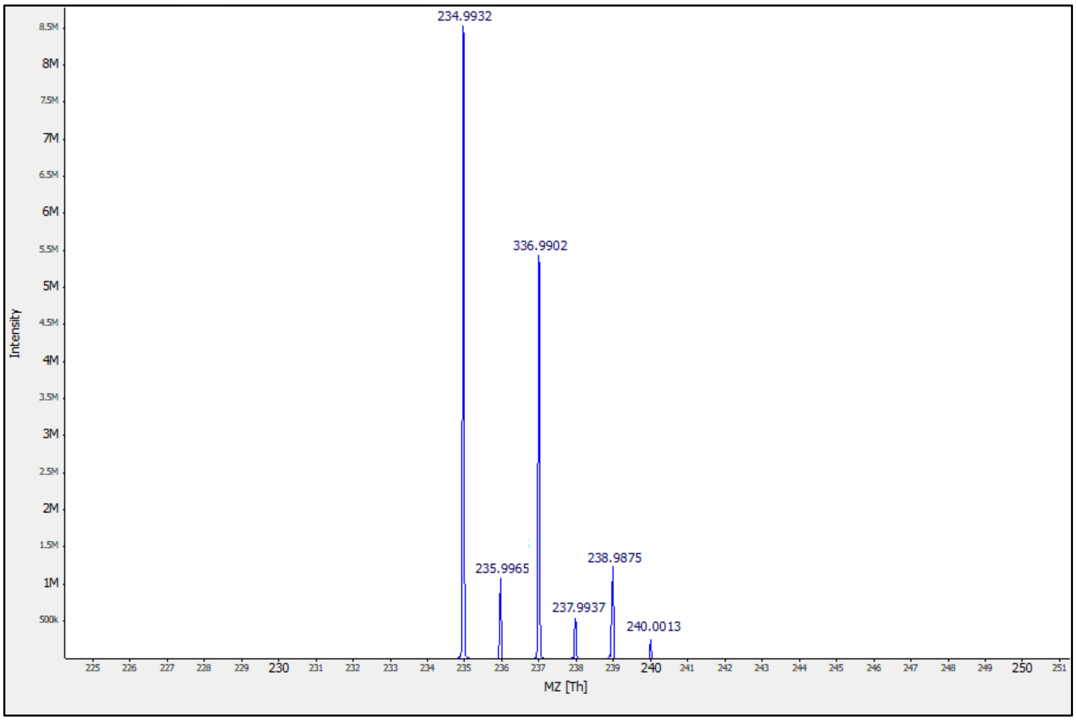

Figure S6. HRESIMS spectrum of 3-(3,5-dichloro-4-hydroxyphenyl)propanoic acid (**1**).

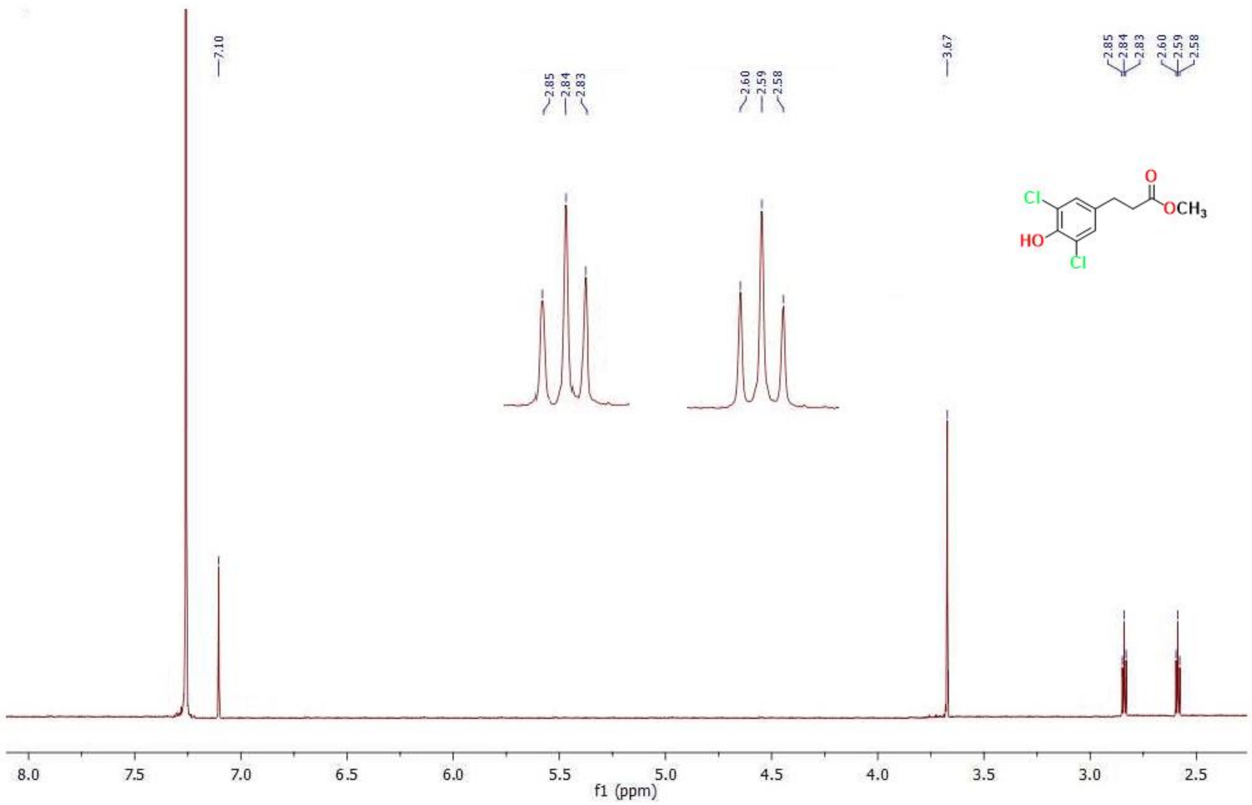

Figure S7. ^{1}H NMR spectrum of 3-(3,5-dichloro-4-hydroxyphenyl)propanoic acid methyl ester (**2**) ($CDCl_3$).

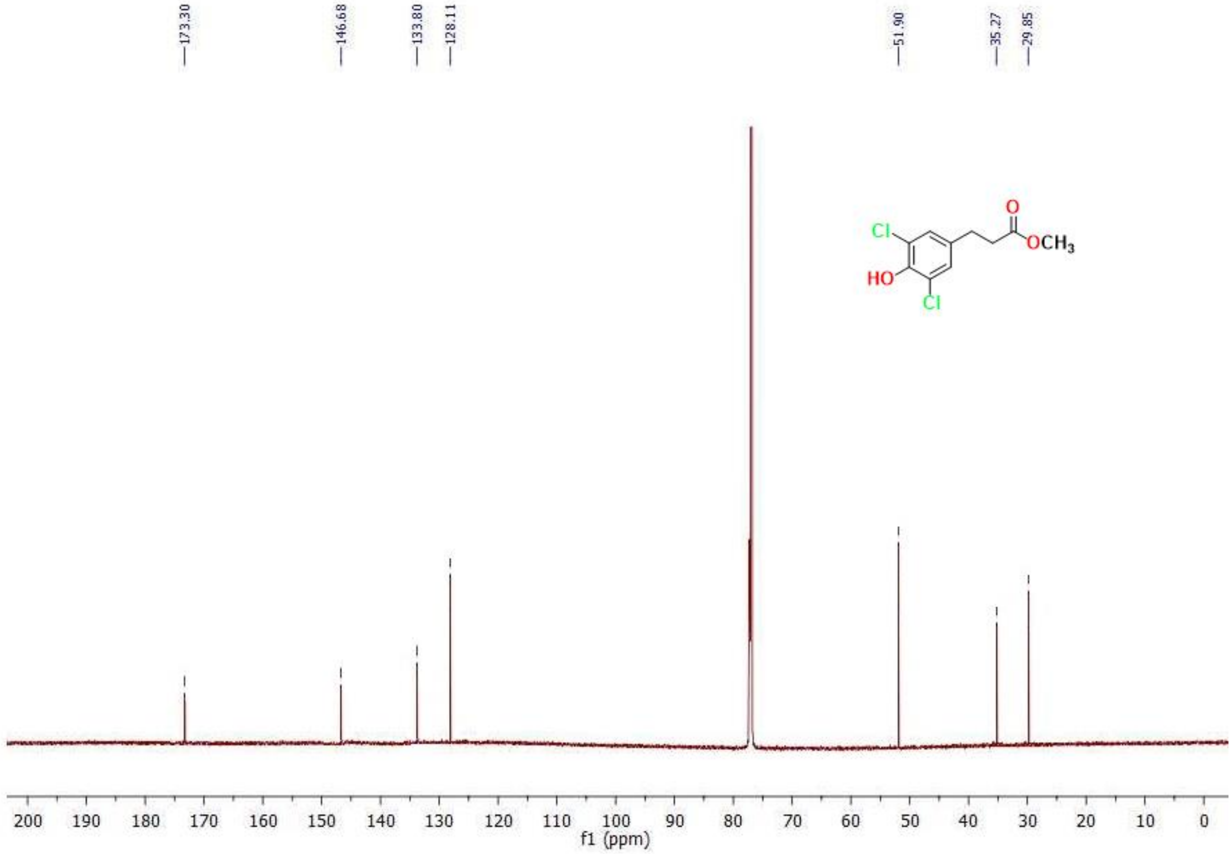

Figure S8. ^{13}C NMR spectrum of 3-(3,5-dichloro-4-hydroxyphenyl)propanoic acid methyl ester (**2**) ($CDCl_3$).

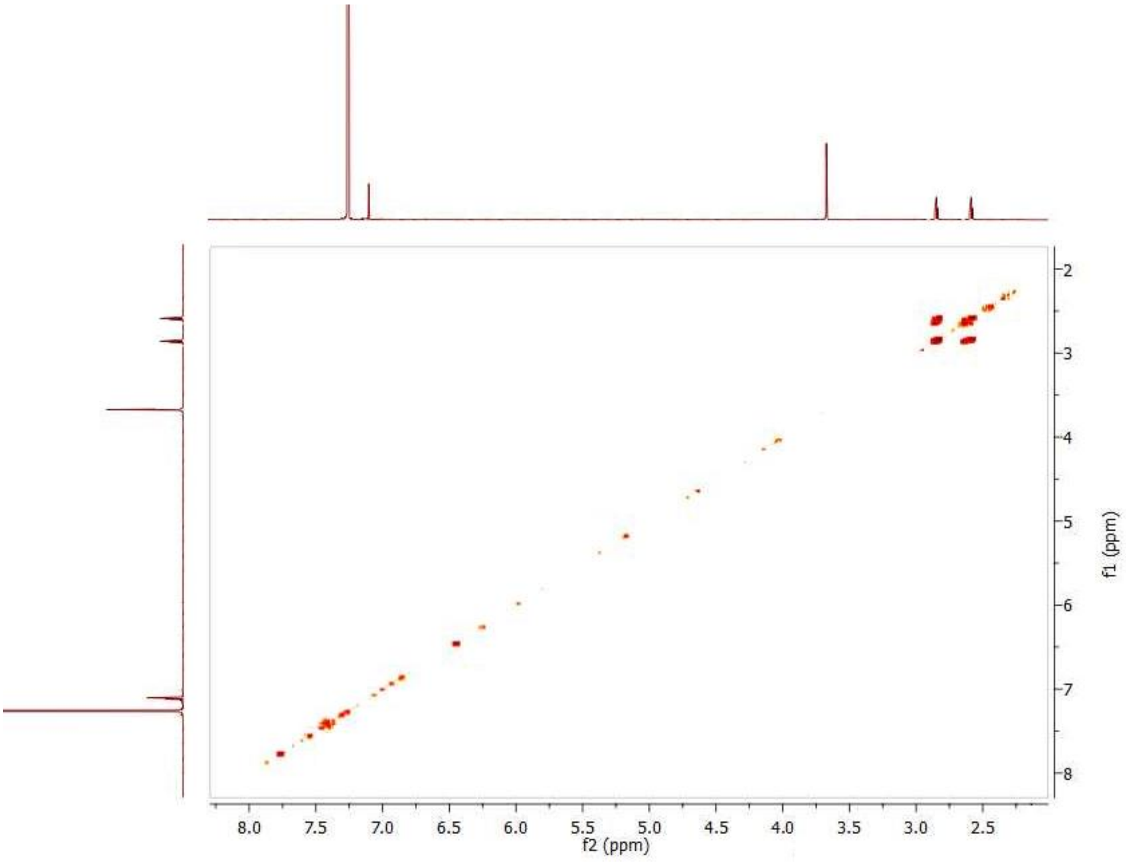

Figure S9. ^{1}H-^{1}H COSY spectrum of 3-(3,5-dichloro-4-hydroxyphenyl)propanoic acid methyl ester (**2**) ($CDCl_3$).

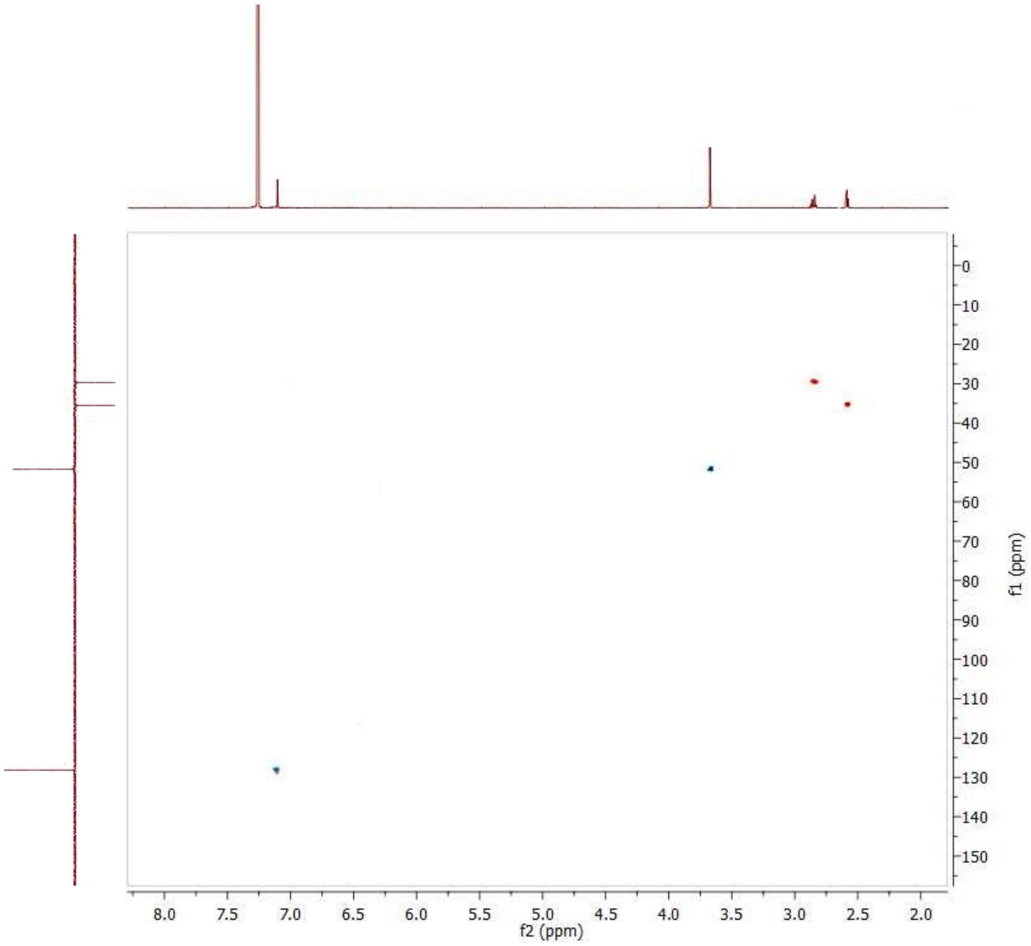

Figure S10. HSQC spectrum of 3-(3,5-dichloro-4-hydroxyphenyl)propanoic acid methyl ester (**2**) ($CDCl_3$).

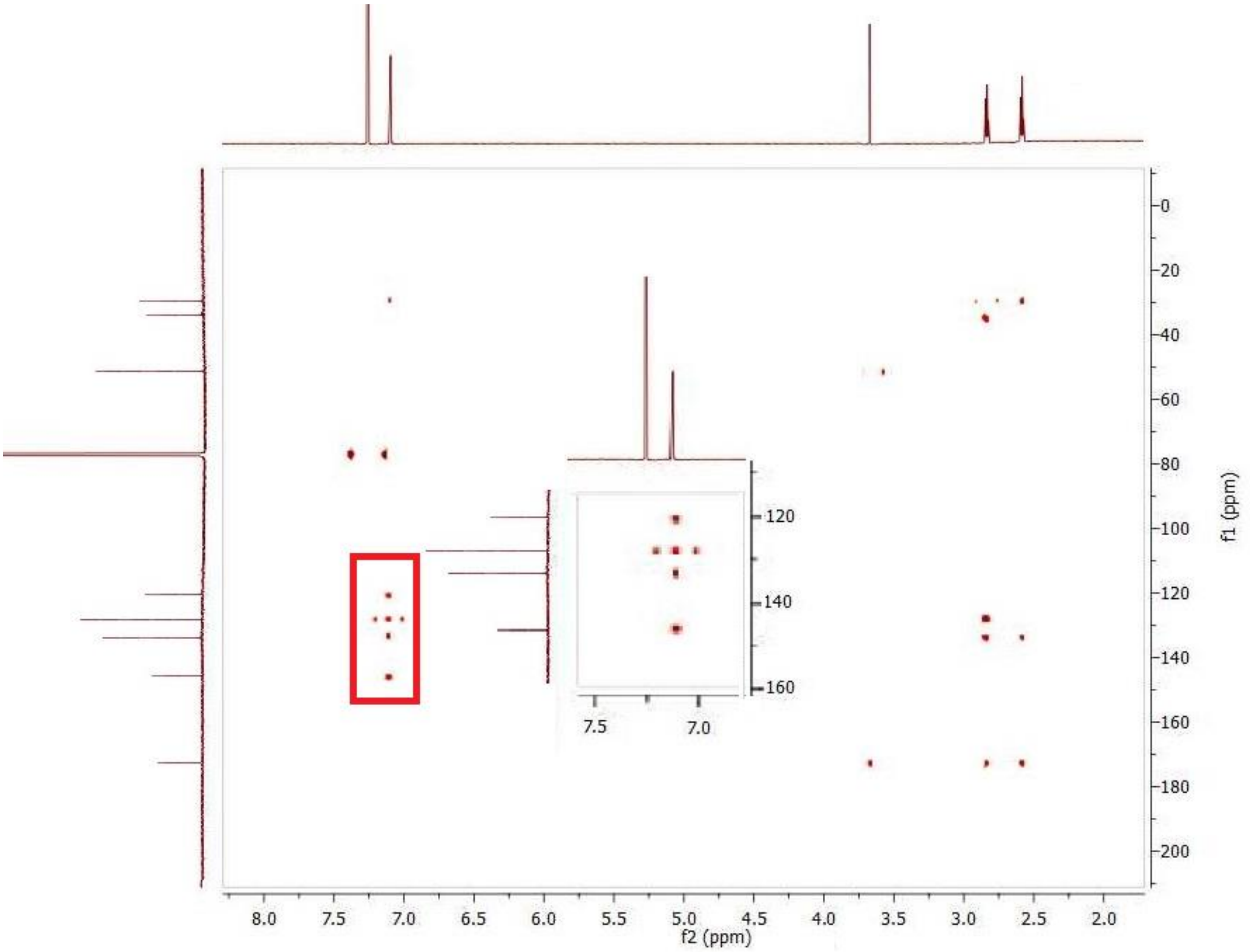

Figure S11. HMBC spectrum of 3-(3,5-dichloro-4-hydroxyphenyl)propanoic acid methyl ester (**2**) ($CDCl_3$).

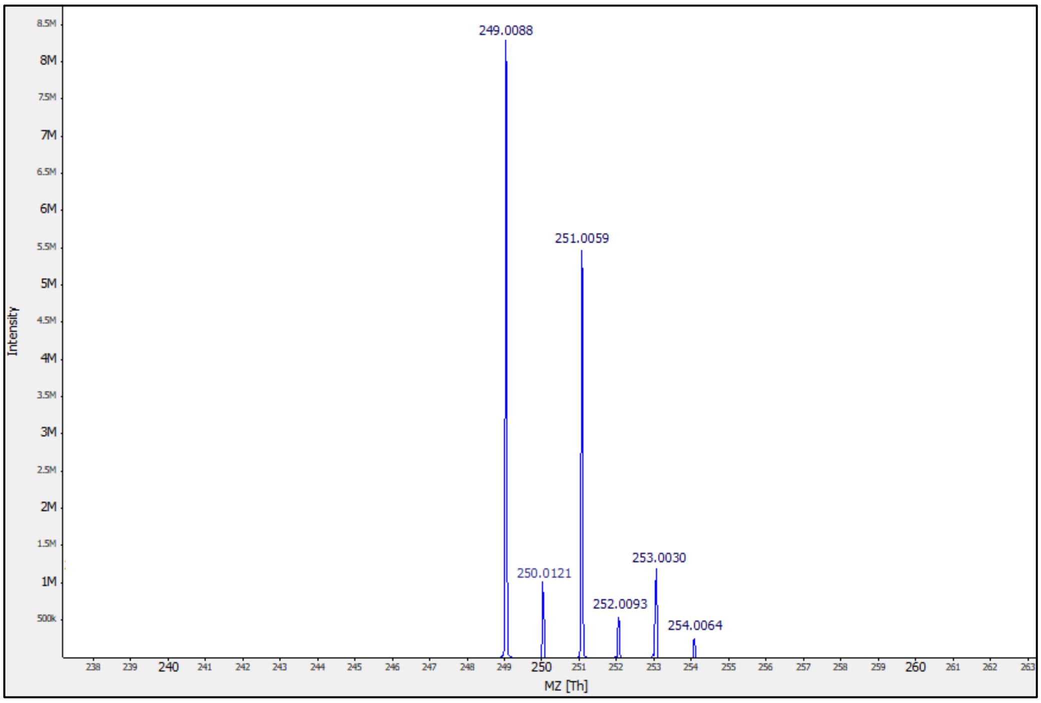

Figure S12. HRESIMS spectrum of 3-(3,5-dichloro-4-hydroxyphenyl)propanoic acid methyl ester (**2**).

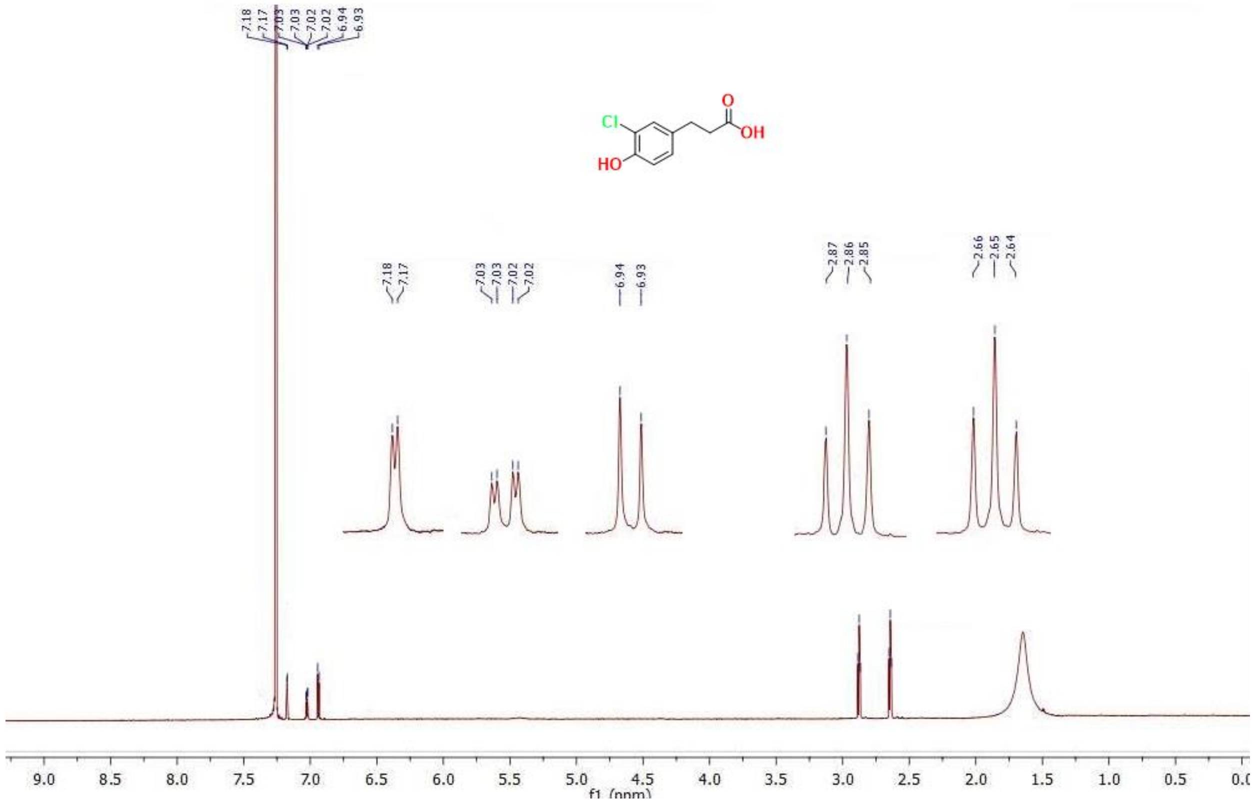

Figure S13. ^{1}H NMR spectrum of 3-(3-chloro-4-hydroxyphenyl)propanoic acid (**3**) ($CDCl_3$).

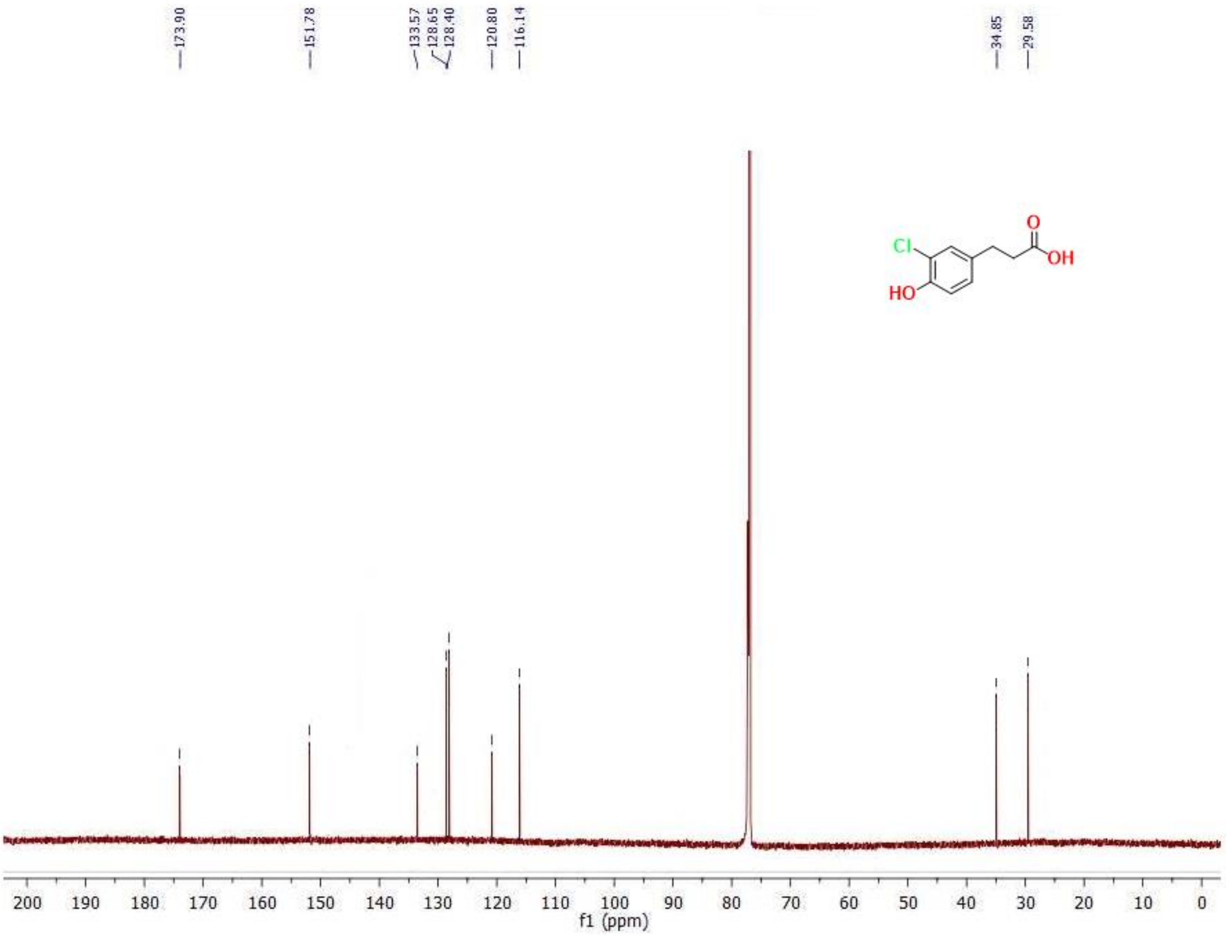

Figure S14. ^{13}C NMR spectrum of 3-(3-chloro-4-hydroxyphenyl)propanoic acid (**3**) ($CDCl_3$).

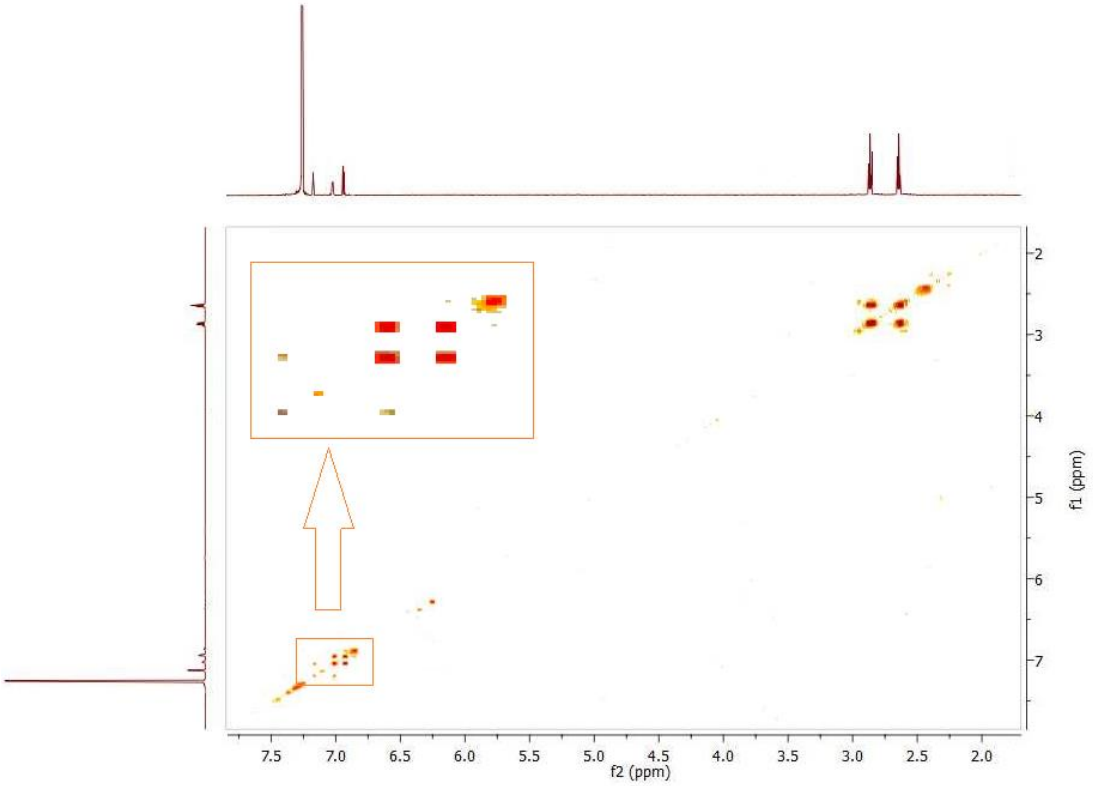

Figure S15. ^{1}H-^{1}H COSY NMR spectrum of 3-(3-chloro-4-hydroxyphenyl)propanoic acid (**3**) ($CDCl_3$).

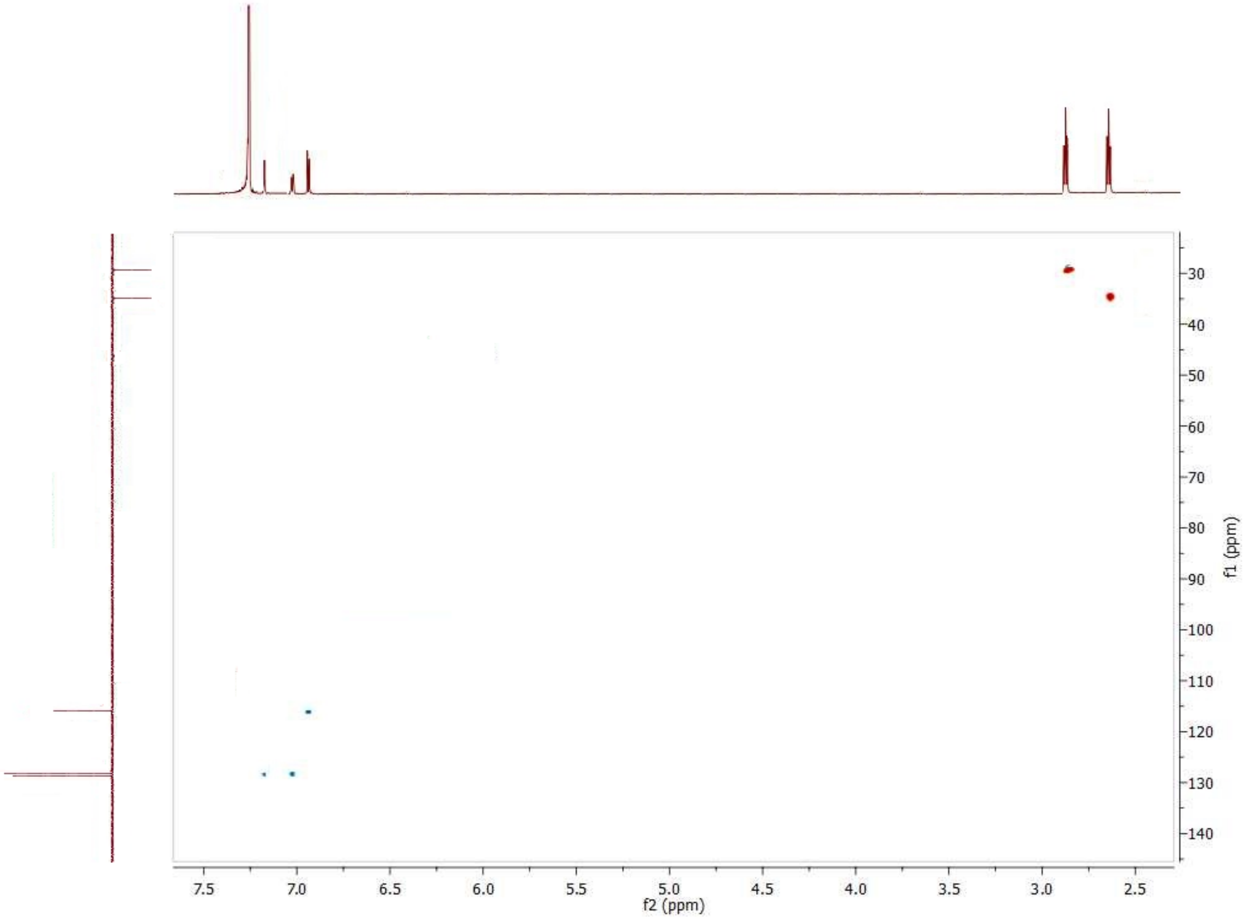

Figure S16. HSQC spectrum of 3-(3-chloro-4-hydroxyphenyl)propanoic acid (**3**) ($CDCl_3$).

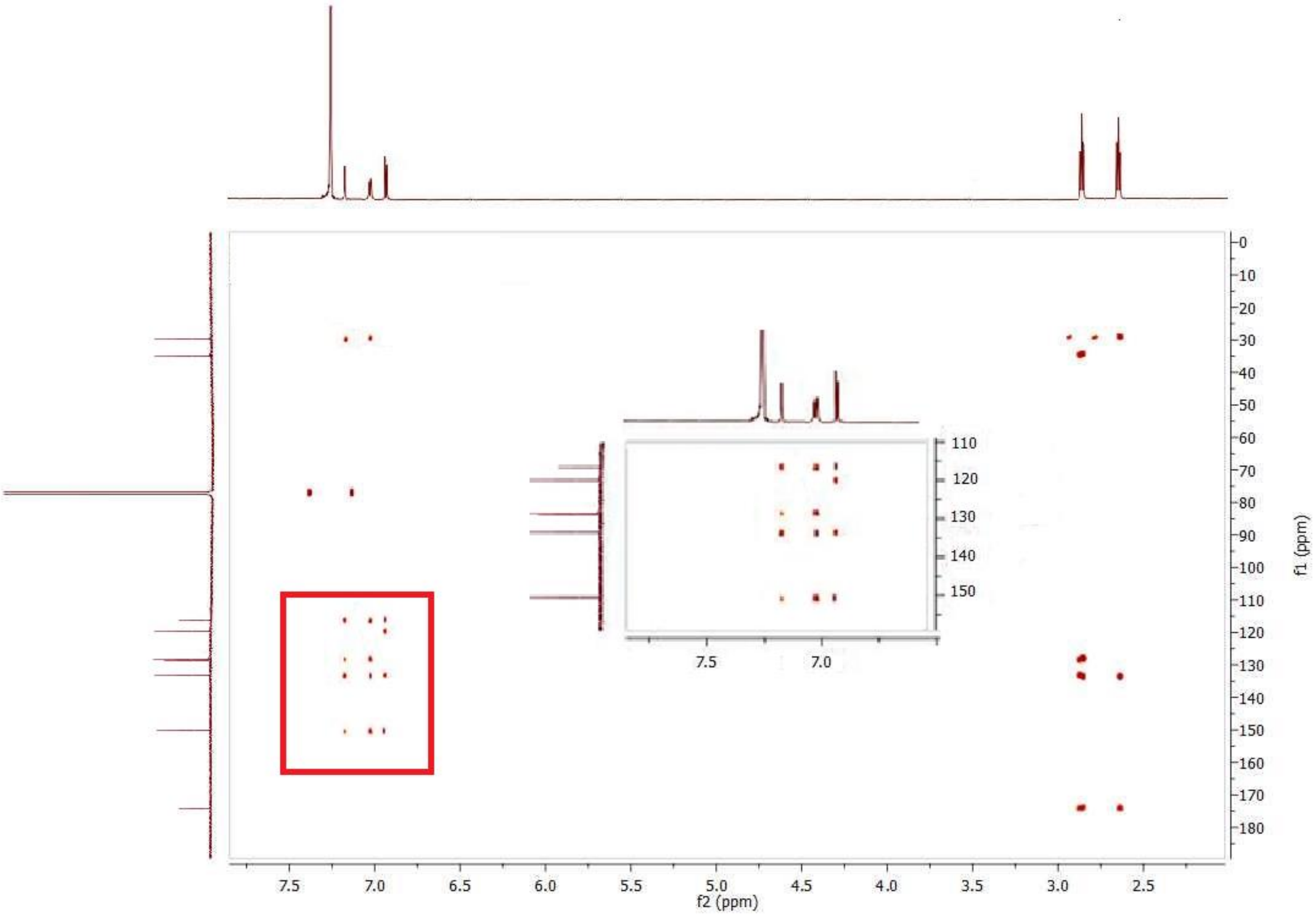

Figure S17. HMBC spectrum of 3-(3-chloro-4-hydroxyphenyl)propanoic acid (**3**) ($CDCl_3$).

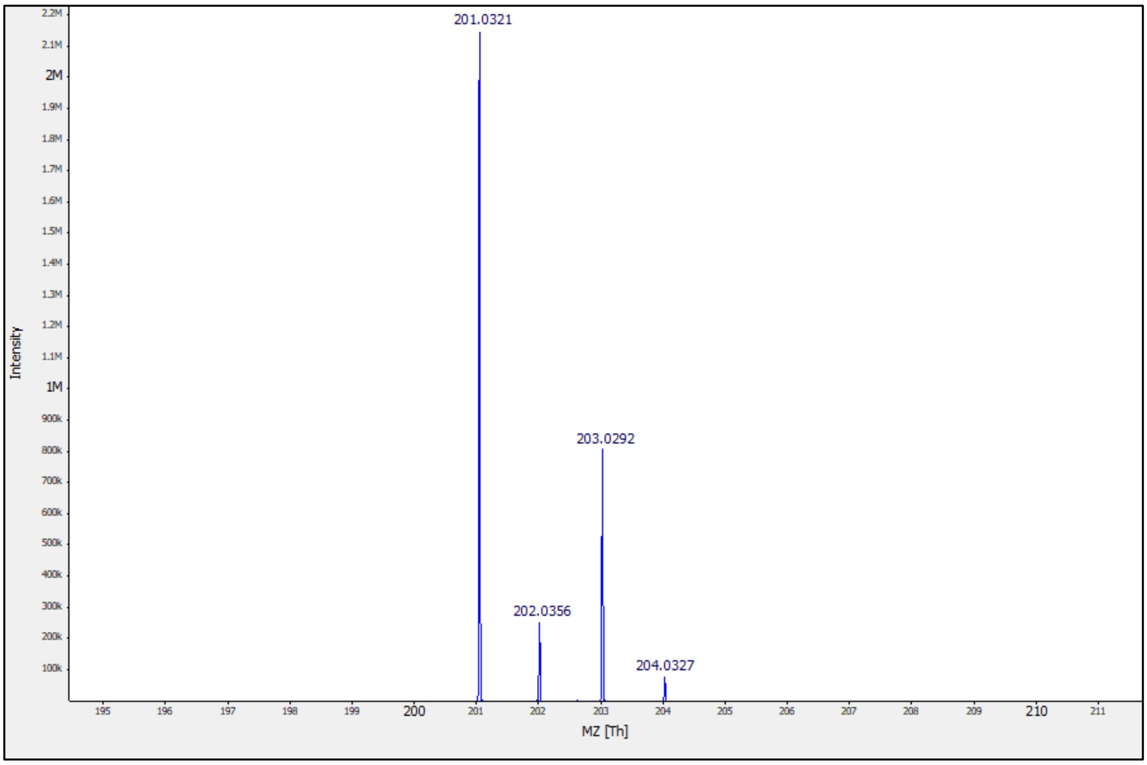

Figure S18. HRESIMS spectrum of 3-(3-chloro-4-hydroxyphenyl)propanoic acid (**3**)

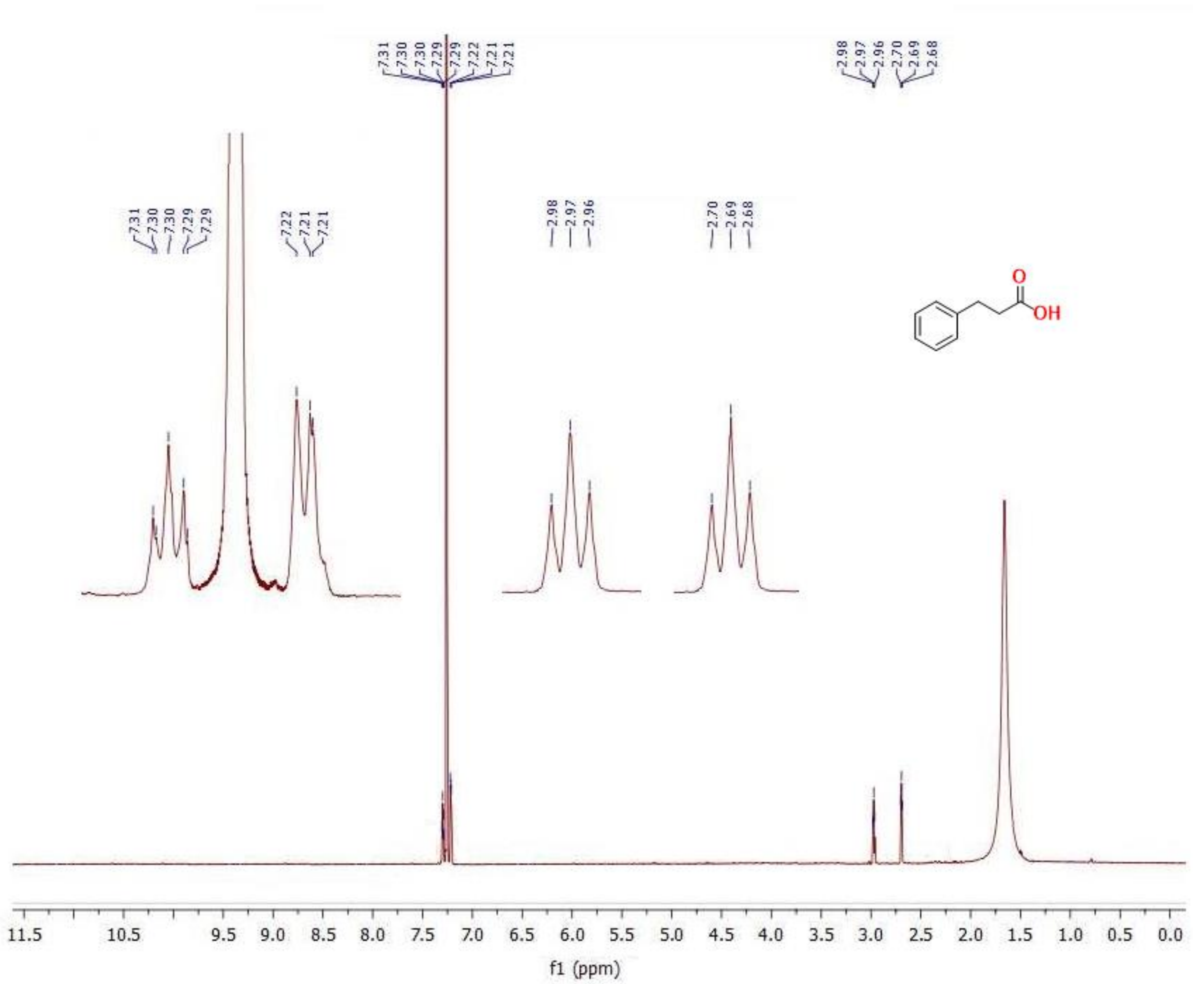

Figure S19. ^{1}H NMR spectrum of 3-phenylpropanoic acid (**4**) (CDCl$_3$).

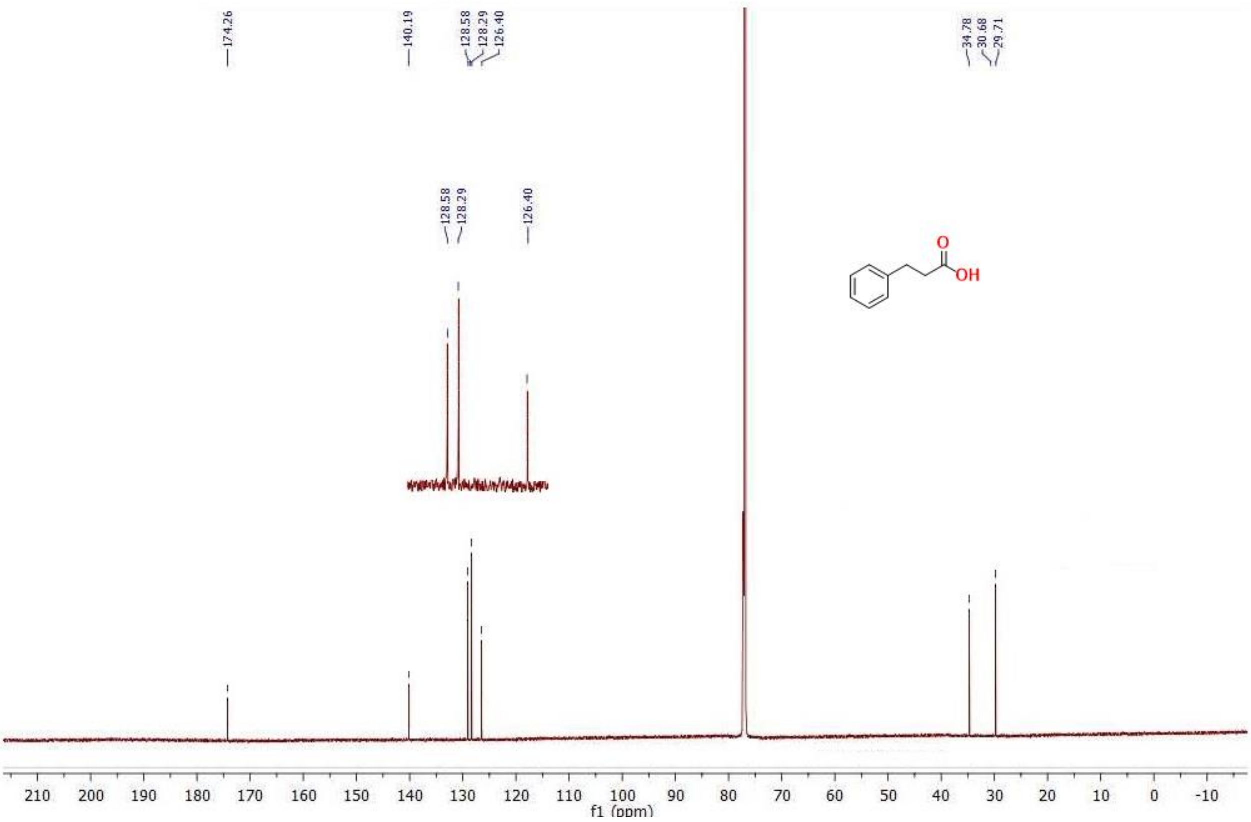

Figure S20. ^{13}C NMR Spectrum of 3-phenylpropanoic acid (**4**) (CDCl$_3$).

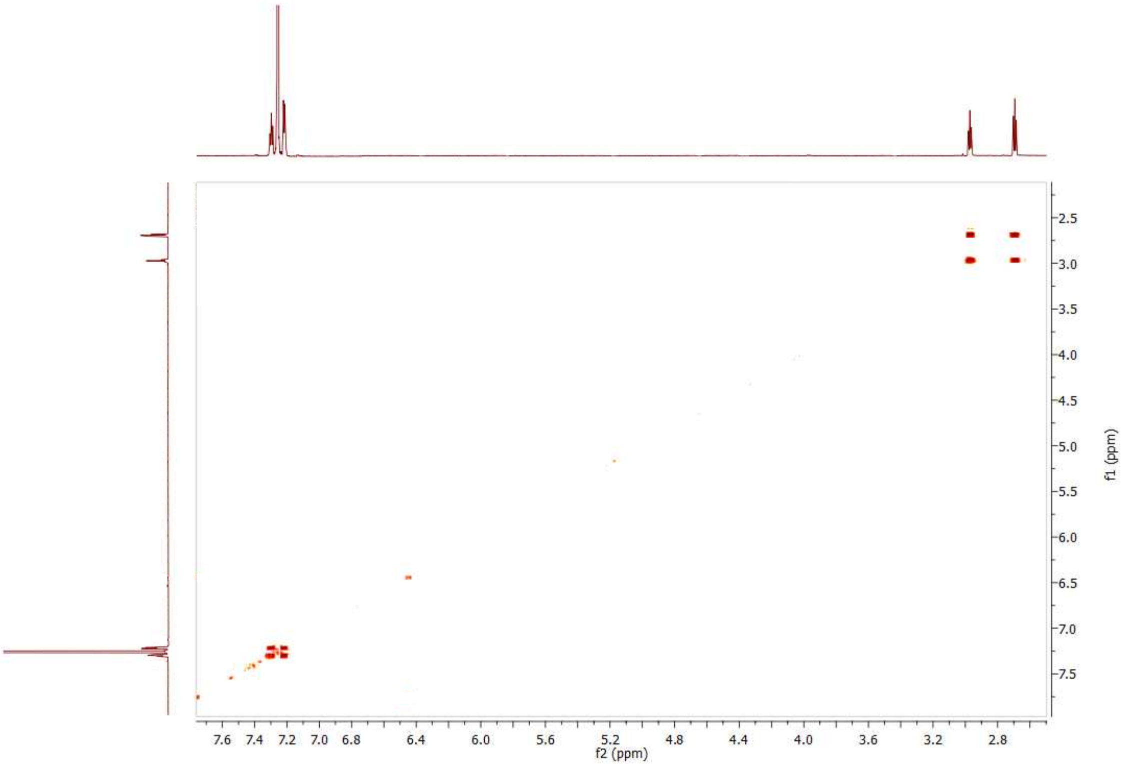

Figure S21. ^1H-^1H COSY spectrum of 3-phenylpropanoic acid (**4**) ($CDCl_3$).

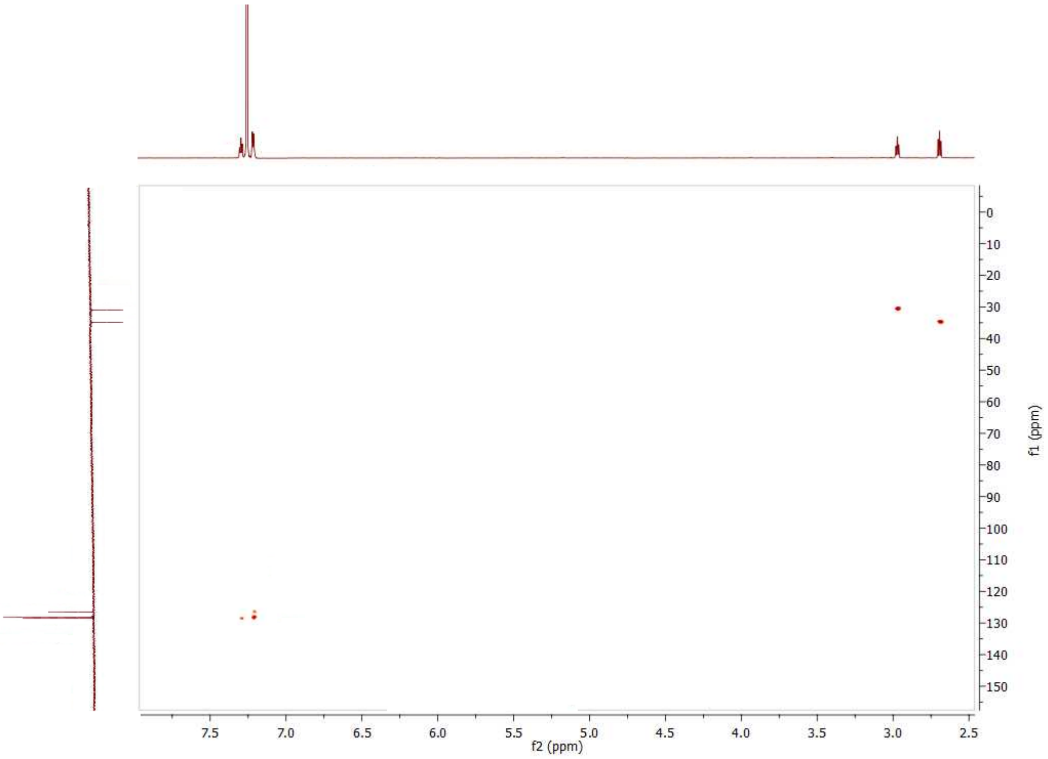

Figure S22. HSQC spectrum of 3-phenylpropanoic acid (**4**) ($CDCl_3$).

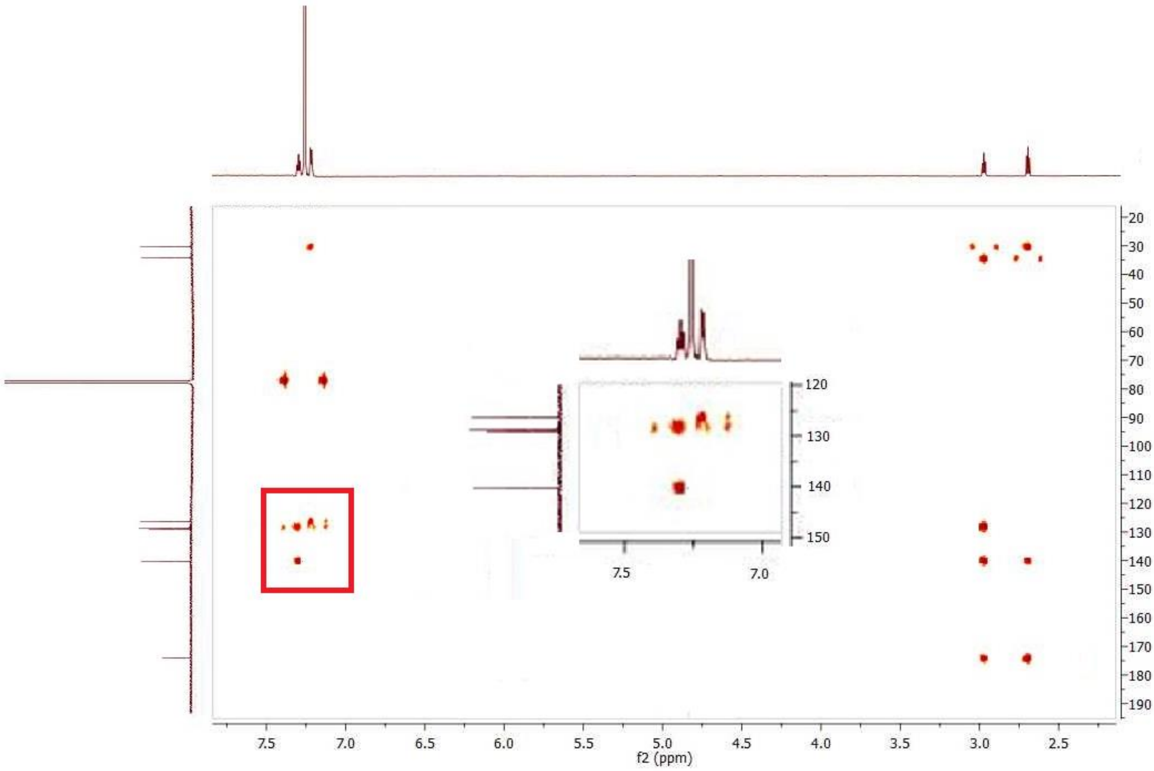

Figure S23. HMBC spectrum of 3-phenylpropanoic acid (**4**) ($CDCl_3$).

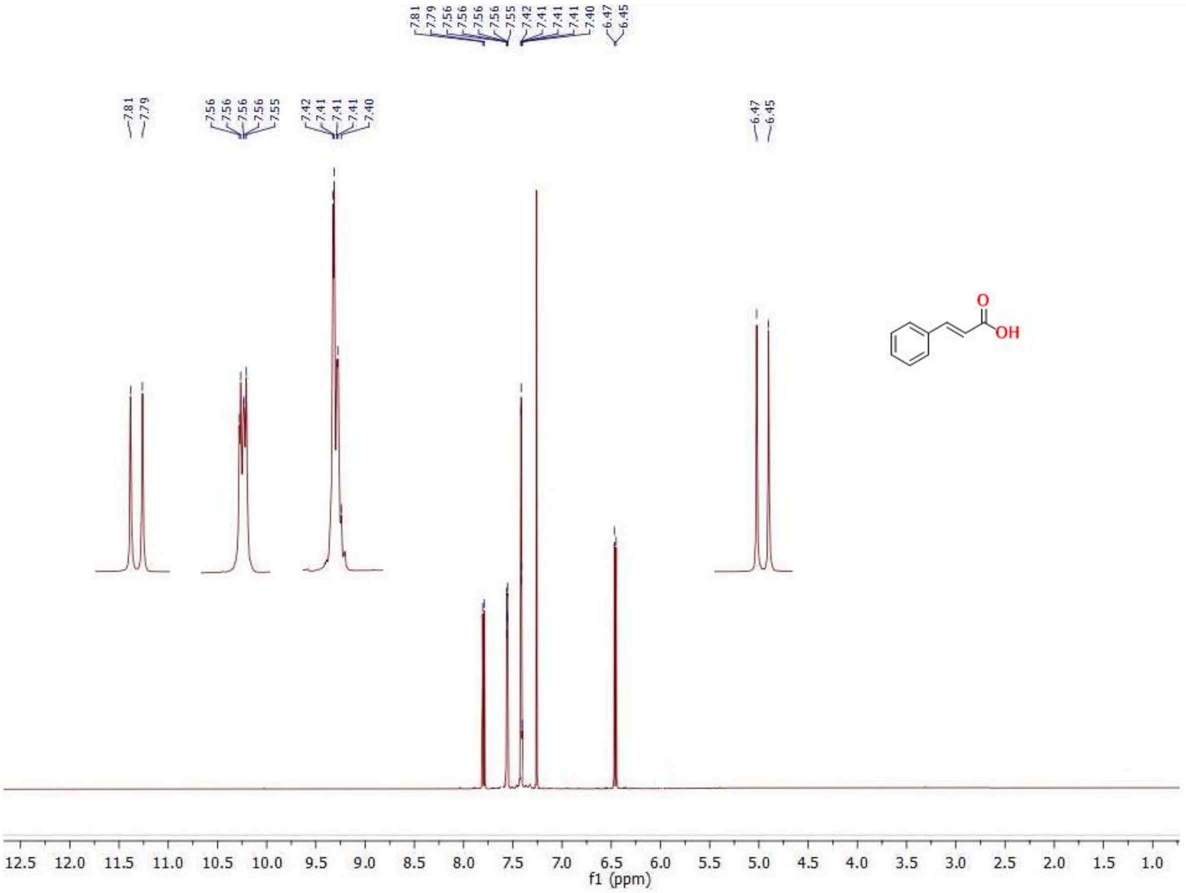

Figure S24. ^1H NMR spectrum of *E*-cinnamic acid (**5**) ($CDCl_3$).

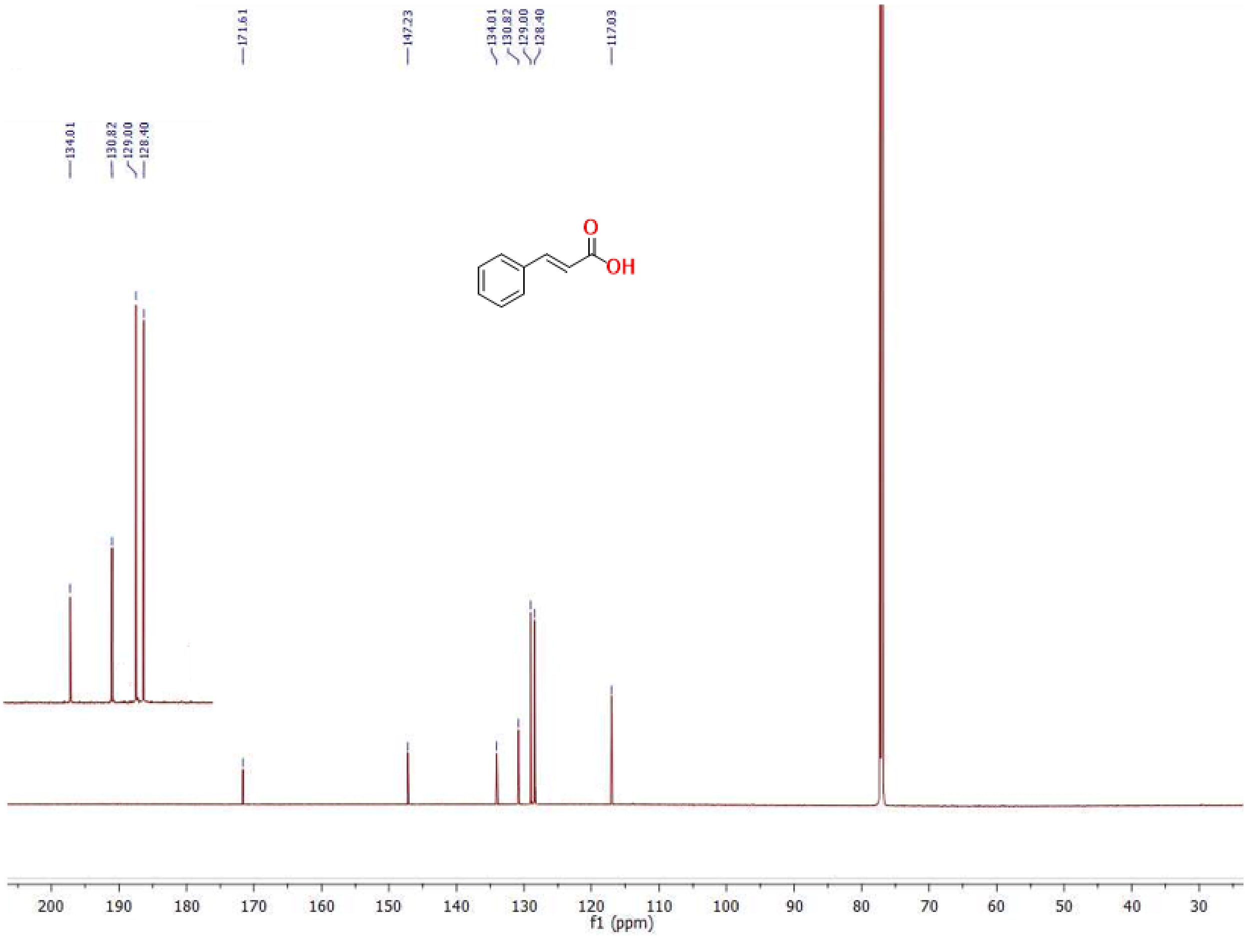

Figure S25. ^{13}C NMR spectrum of *E*-cinnamic acid (**5**) ($CDCl_3$).

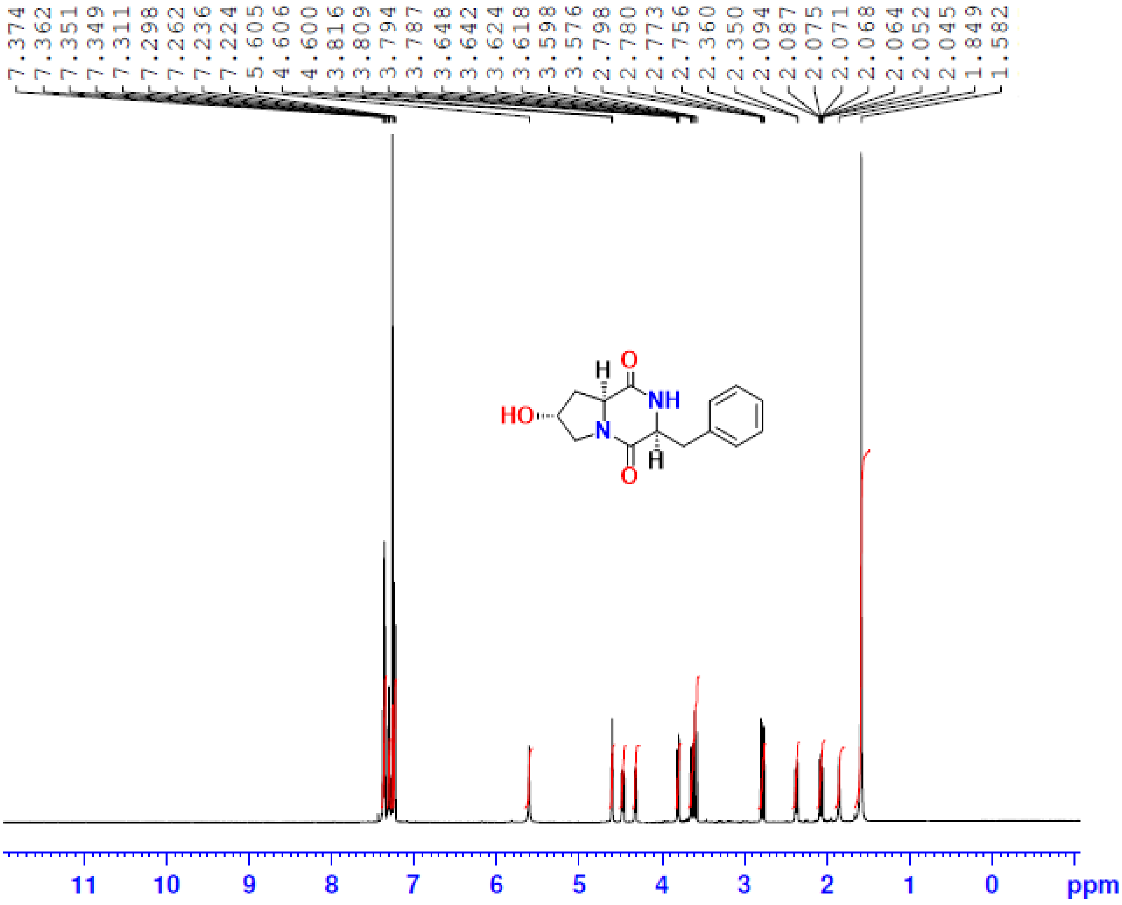

Figure S26. ^{1}H NMR spectrum of cyclo(L-Phe-*trans*-4-OH-L-Pro) (**6**) (CDCl$_3$).

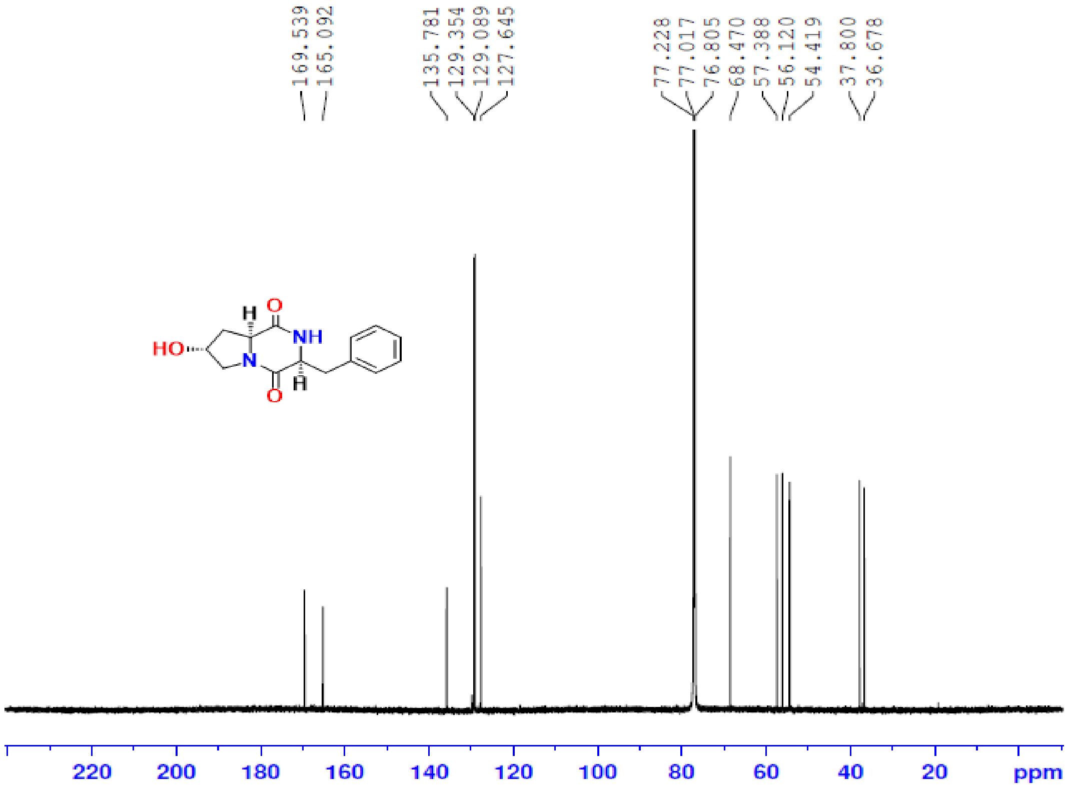

Figure S27. ^{13}C NMR spectrum of cyclo(L-Phe-*trans*-4-OH-L-Pro) (**6**) (CDCl$_3$).

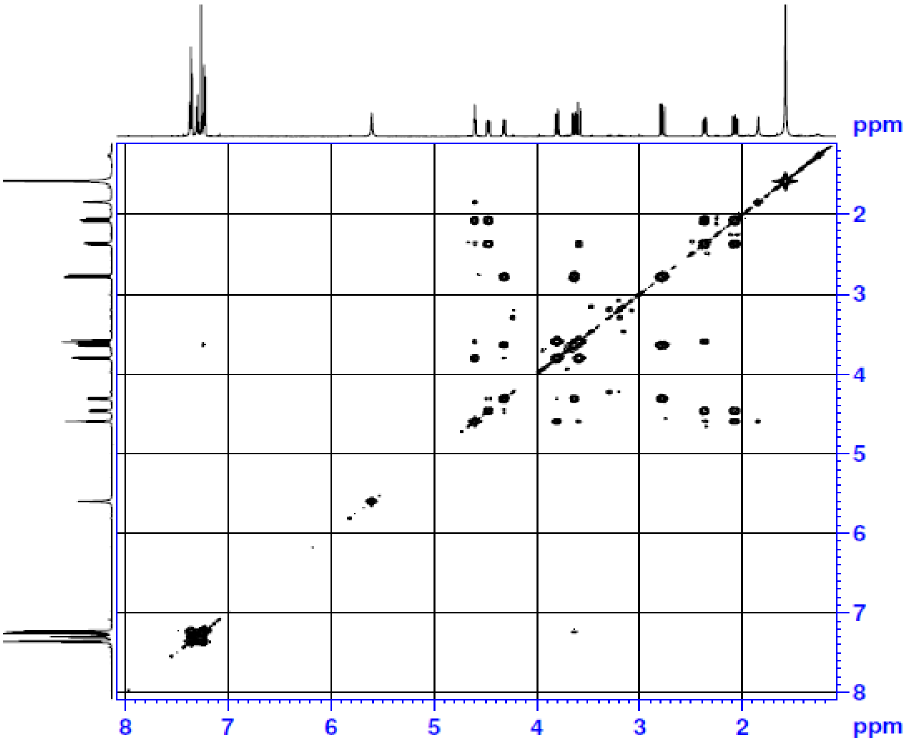

Figure S28. ^{1}H-^{1}H COSY spectrum of cyclo(L-Phe-*trans*-4-OH-L-Pro) (**6**) (CDCl$_3$).

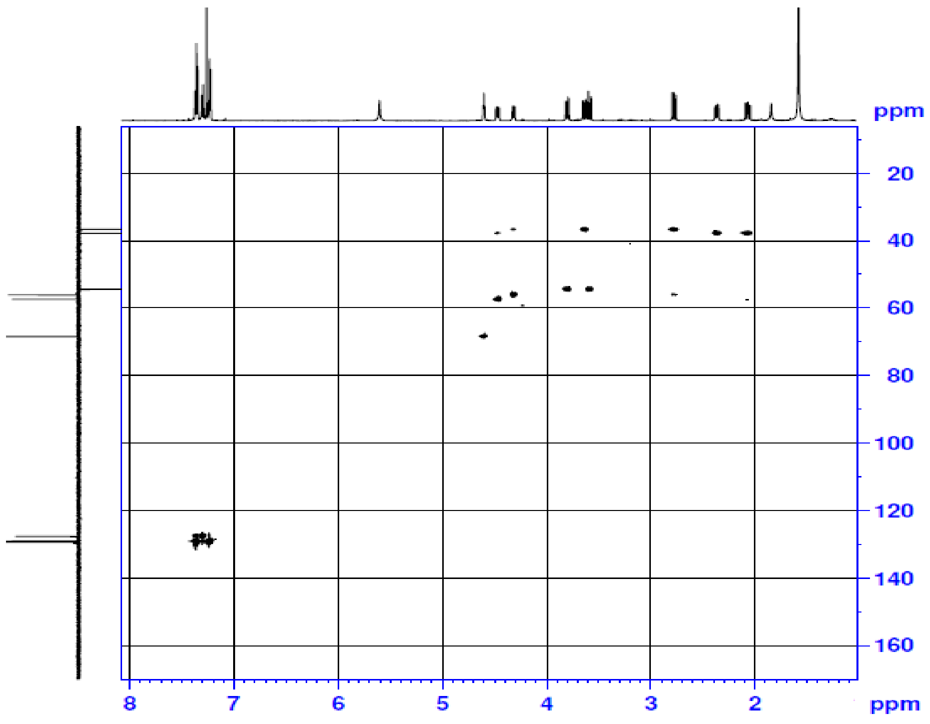

Figure S29. HSQC spectrum of cyclo(L-Phe-*trans*-4-OH-L-Pro) (**6**) ($CDCl_3$).

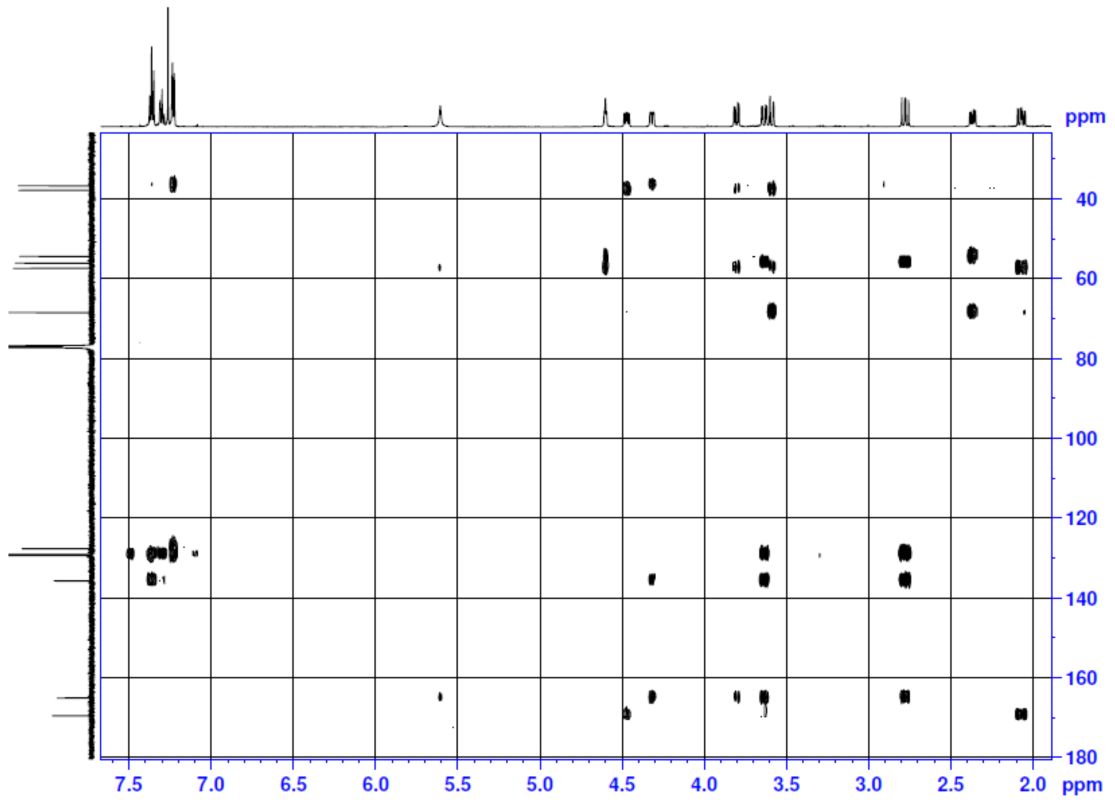

Figure S30. HMBC spectrum of cyclo(L-Phe-*trans*-4-OH-L-Pro) (**6**) ($CDCl_3$).

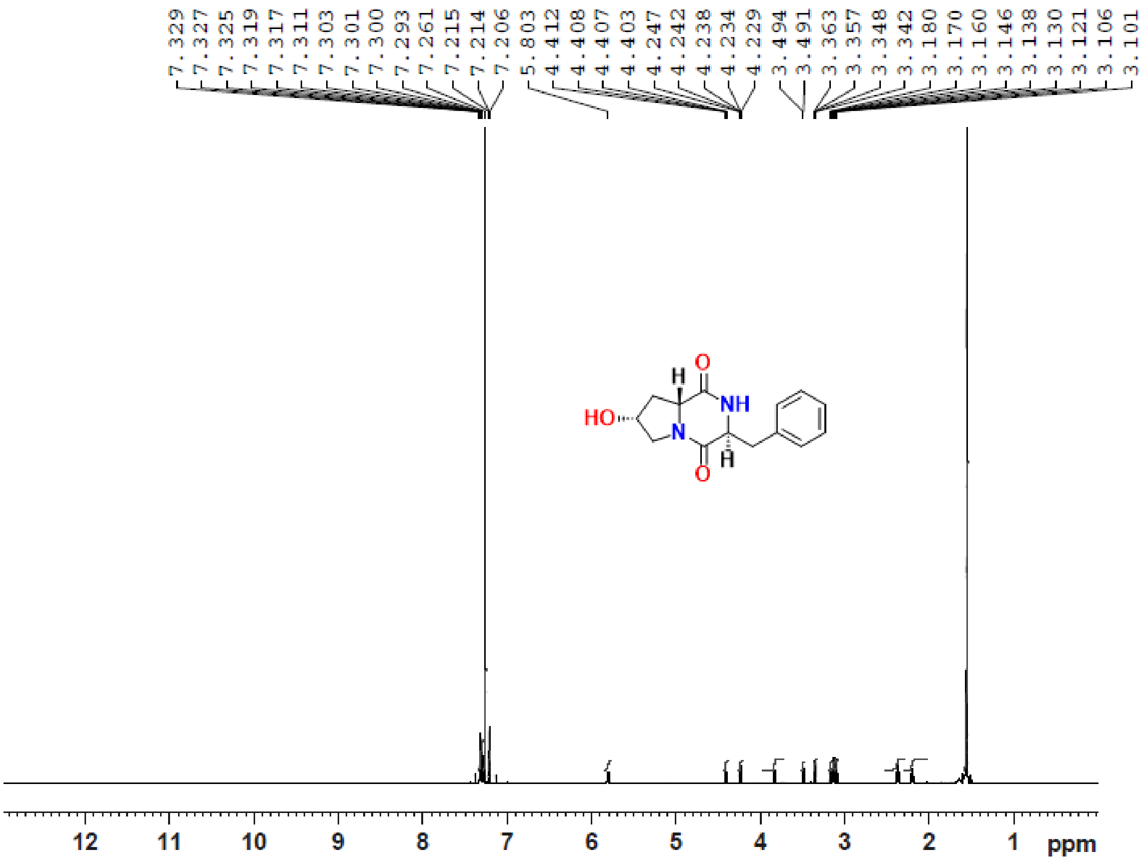

Figure S31. ^{1}H NMR spectrum of cyclo(L-Phe-*cis*-4-OH-D-Pro) (**7**) ($CDCl_3$).

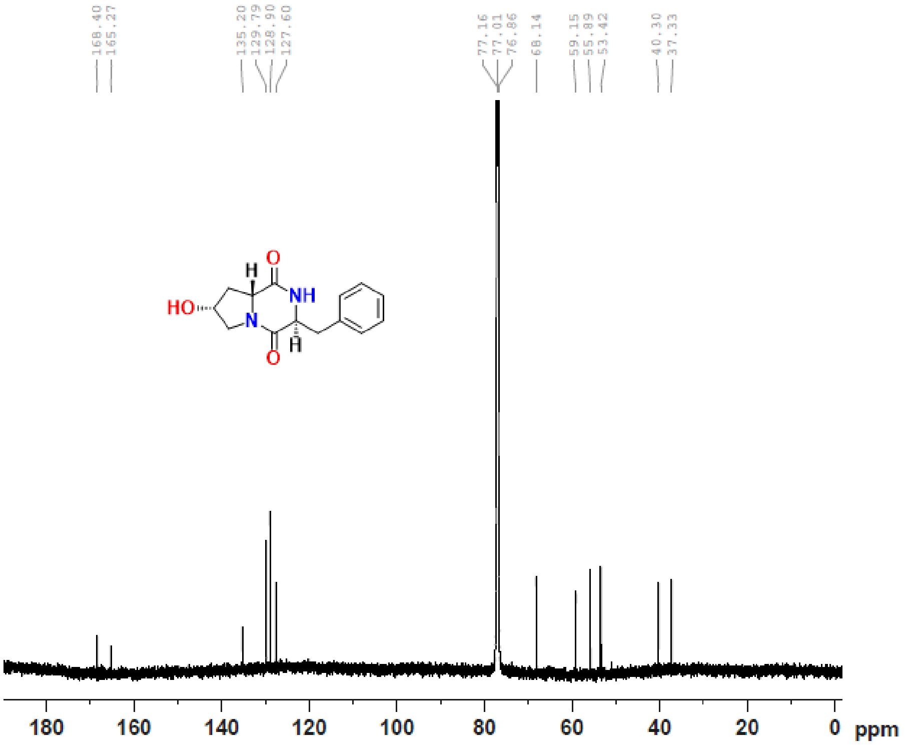

Figure S32. ^{13}C NMR spectrum of cyclo(L-Phe-*cis*-4-OH-D-Pro) (**7**) ($CDCl_3$).

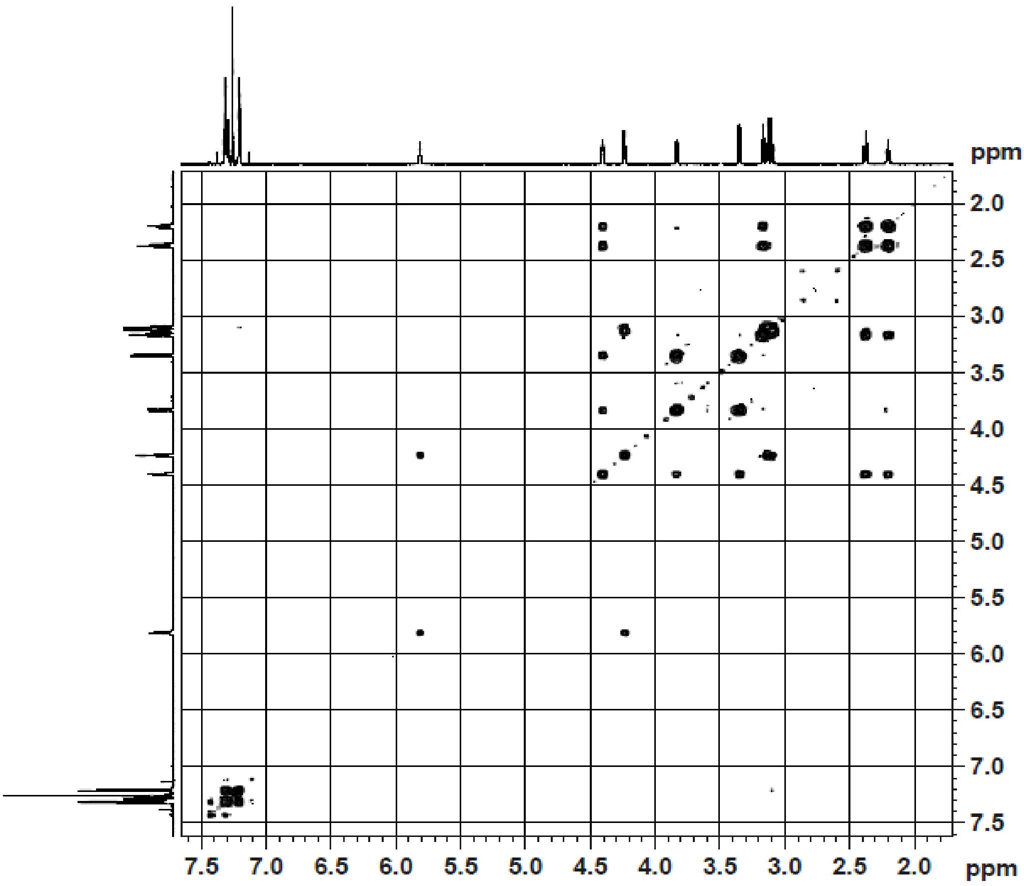

Figure S33. ^1H-^1H COSY spectrum of cyclo(L-Phe-*cis*-4-OH-D-Pro) (**7**) ($CDCl_3$).

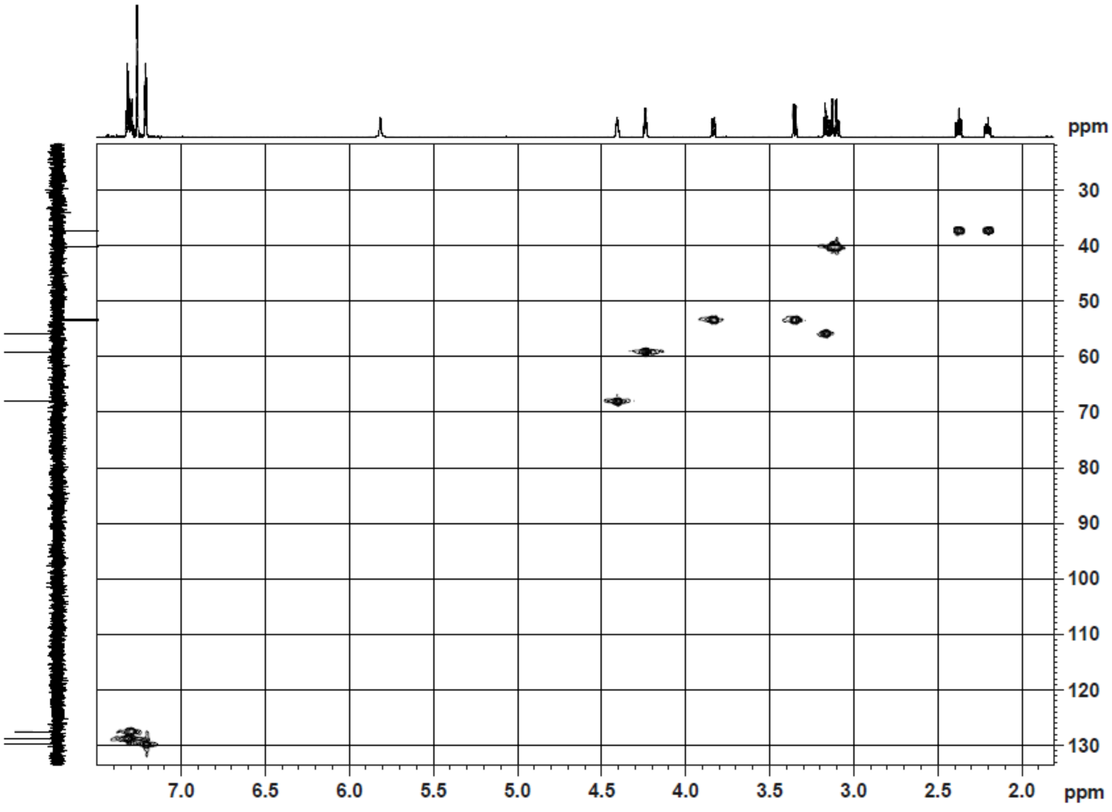

Figure S34. HSQC spectrum of cyclo(L-Phe-*cis*-4-OH-D-Pro) (**7**) ($CDCl_3$).

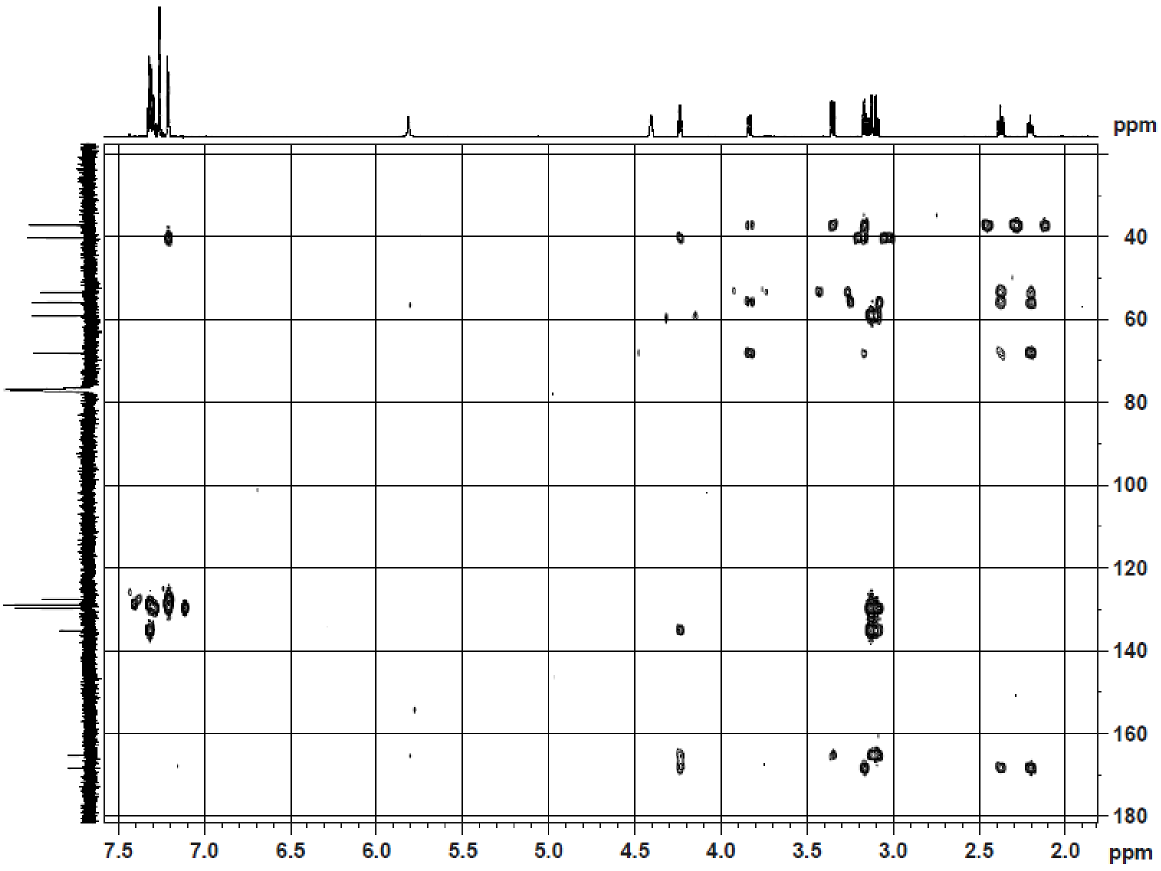

Figure S35. HMBC spectrum of cyclo(L-Phe-*cis*-4-OH-D-Pro) (**7**) ($CDCl_3$).
